# Disruption of methionine metabolism drives erythroid cell fate reprogramming by remodeling the H3K4me3 landscape

**DOI:** 10.1172/JCI203565

**Published:** 2026-07-14

**Authors:** Lei Sun, Hengchao Zhang, Mengjia Li, Quande Lin, Xiuyun Wu, Ying Cheng, Shihui Wang, Yan Hou, Yaomei Wang, Yue Sheng, Jing Liu, Xiuli An, Ting Wang, Lixiang Chen

**Affiliations:** 1 Aging Decoding and Regeneration Institute, School of Life Sciences, Zhengzhou University, Zhengzhou, China.; 2 Department of Hematology, First Affiliated Hospital of Zhengzhou University, Zhengzhou, China.; 3 Henan Cancer Hospital, Affiliated Cancer Hospital of Zhengzhou University, China.; 4 Institute of Hematology, People’s Hospital of Zhengzhou University, ZhengZhou, China.; 5 Department of Hematology, the Second Xiangya Hospital, Central South University, Changsha, China.; 6 Molecular Biology Research Center & Center for Medical Genetics, School of Life Sciences, Central South University, Changsha, China.; 7 Laboratory of Membrane Biology, New York Blood Center, New York, New York USA.

**Keywords:** Cell biology, Hematology, Epigenetics

## Abstract

The methionine cycle plays critical roles in cell fate determination by shaping epigenetic landscape, yet its function in human erythropoiesis remains undefined. Here, we show that disruption of methionine metabolism by compromising the key enzyme adenosylhomocysteinase (AHCY) reshapes H3K4me3 landscape, causing erythroid cell fate reprogramming. AHCY deficiency severely impaired erythroid differentiation and expansion, leading to the generation of nonerythroid lineage hematopoietic cells, including stem/progenitor cells and immune cells, as evidenced by single-cell RNA-seq, and pseudo temporal analysis delineated a precise dedifferentiation trajectory, revealing erythroblasts transitioning back to MEPs and HSCs. Moreover, the human hematopoietic system could be reconstituted in the immunodeficient NCG-X mice by transplanting AHCY-deficient erythroblasts. Mechanistically, AHCY deficiency reduced global H3K4me3 levels and altered its genomic distribution, resulting in the upregulated expression of nonerythroid transcription factors and downregulated expression of erythrocyte lineage-specific transcription factors. Integrated single-cell analyses identified transitional states with diminished AHCY in the erythroblasts of a patient with acute myeloid leukemia (AML). Further flow cytometry confirmed the reduced H3K4me3 level in patient-derived erythroid cells. Erythroblasts isolated from patients with AML with reduced H3K4me3 exhibited a dedifferentiation potential into progenitor-like states. Our findings reveal a metabolic-epigenetic axis governing cell fate reprogramming in human erythropoiesis and provide insights into leukemia-associated anemia.

## Abstract 2

Disruption of methionine metabolism by AHCY deficiency remodels H3K4me3 landscape and drives erythroid cell fate reprogramming in human erythropoiesis.

## Graphical abstract

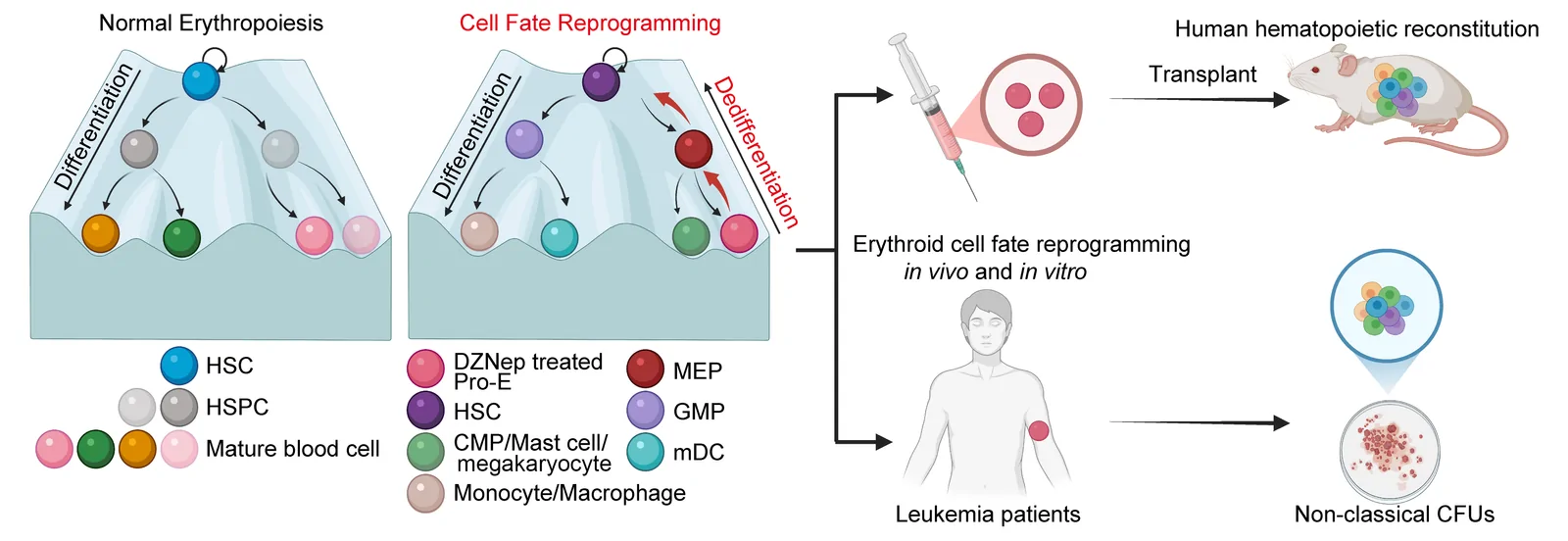

## Introduction

Methionine metabolism fuels methylation reactions by generating S-adenosylmethionine (SAM), the universal methyl donor for DNA, RNA, histones, and nonhistone proteins (1–4). The demethylation of SAM generates S-adenosylhomocysteine (SAH), which is hydrolyzed by adenosylhomocysteinase (AHCY). As a structural analog of SAM, SAH acts as a potent feedback inhibitor of SAM-dependent methyltransferases (5). Disruption of methionine metabolism via methionine depletion or AHCY deficiency shifts the SAM/SAH ratio and leads to aberrant DNA and histone methylation (6–9). Methionine metabolism orchestrates cell fate decisions by linking metabolic state to epigenetic modification. This is evidenced by the AHCY inhibitor 3-Deazaneplanocin A (DZNep) (10, 11), which promotes pluripotency by reducing repressive DNA and H3K9 methylation at loci such as OCT4 (12). In multiple reprogramming contexts, including from extraembryonic endoderm-like states and in human pluripotent stem cells, DZNep has been identified as a core epigenetic regulator that reactivates pluripotency genes like OCT4 (13, 14). Similarly, methionine deprivation in human ESCs/iPSCs reduces DNA/histone methylation, dampens NANOG expression, and enhances differentiation (15).

Erythropoiesis is a multistep process comprising 3 major phases, termed early erythropoiesis, terminal erythroid differentiation, and reticulocyte maturation (16, 17). Erythroid lineage progression is marked by distinct morphological and functional changes at each developmental stage. (18–22). Precise regulation of lineage- and stage-specific gene expression, governed by DNA methylation and histone methylation, is pivotal for mammalian erythropoiesis. Genome-wide DNA methylation analysis from a number of studies have shown a progressive, global demethylation during erythropoiesis (23–25). Our and others’ prior studies demonstrated that deficiencies of DNA methyltransferases (DNMTs) and Ten-eleven translocation (TET) enzymes impair human erythropoiesis in the stage-dependent manner (26–28). Disruption of DNA methylation landscape upon loss of DNMT3A and TET2 resulted in specific erythroid or myelomonocytic lineage skewing phenotypes by tuning the expression of lineage-determining erythroid transcription factors (TFs) (29). Histone methylations also were shown to regulate cell fate determination during erythropoiesis. PRMT6, recruited by RUNX1, forms a SIN3A-HDAC1 corepressor complex, promoting erythroid fate by repressing megakaryopoiesis genes (30). High H3R2me2a and low H3K4me3 at erythroid-specific TF promoters correlate with megakaryocytic bias from megakaryocyte-erythroid progenitors (MEPs) (30, 31). Deletion of NSD1, a key H3K36 methylase, impairs GATA1-regulated erythroid differentiation and induces erythroleukemia (32, 33). Collectively, these findings underscore the vital role of methylation-dependent epigenetic modification in erythropoiesis by determining cell fate. However, as the primary source of methyl groups, the contribution of methionine metabolism in erythropoiesis remains largely unknown.

Here, we identify AHCY as a key regulator of human erythropoiesis, directing cell fate decisions by modulating the H3K4me3 epigenetic landscape. Our findings uncover a novel mechanism by which methionine metabolism controls erythropoiesis through methylation-dependent epigenetic modifications. Importantly, we demonstrate that the erythroid cell fate reprogramming, driven by altered H3K4me3 landscape, contributes to the pathogenesis of leukemia-associated anemia, highlighting a pivotal role for epigenetic dysregulation in this condition.

## Results

### Deficiency of AHCY impairs human erythropoiesis.

**Figure 1:**
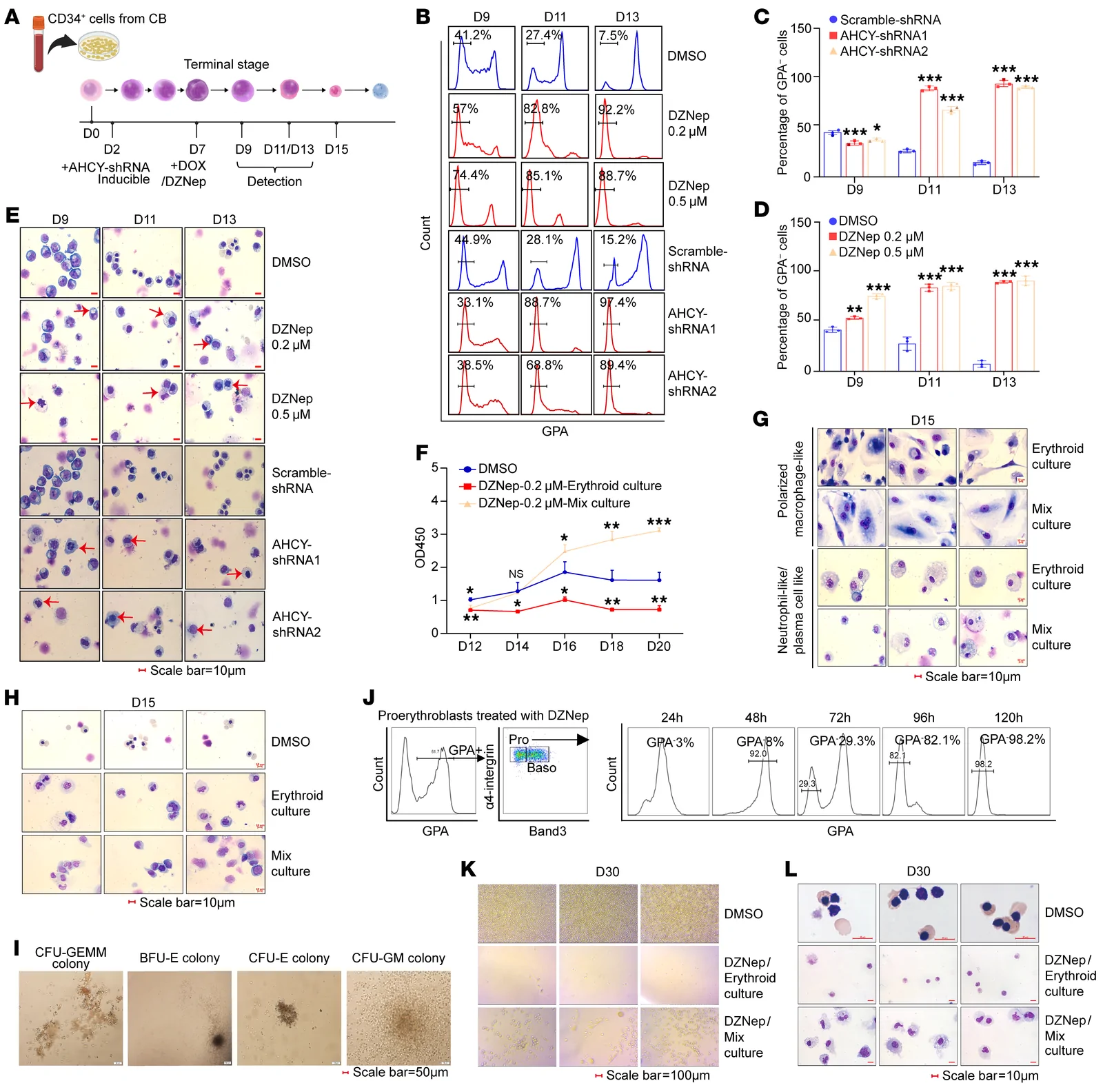
AHCY deficiency of erythroblast results in the generation of nonerythroid lineage hematopoietic cells. (**A**) Schematic diagram of experimental outline for the strategy to achieve AHCY deficiency. Day 0: CD34^+^ cells obtained. Lentivirus transduction at day 2. DOX or DZNep added at day 7. (**B**) Flow cytometry analysis showing the percentage of GPA^–^ cells on days 9, 11, and 13. (**C** and **D**) Quantitative analysis of GPA^–^ cells of Scramble-shRNA, AHCY-shRNA, DMSO, and DZNep (0.2 μM, 0.5 μM) treated group. (**E**) Cytospin images of cells from Scramble-shRNA, AHCY-shRNA, and DMSO and DZNep (0.2 μM, 0.5 μM) treated groups on days 9, 11 and 13, red arrow indicates cells with nonerythroid lineage morphology. Scale bar: 10 μm. (**F**) Growth curves of DMSO and DZNep-treated cells that were cultured in erythroid or mixed culture medium. (**G**) Cytospin images of cell climbing sheets of DZNep-treated cells cultured in erythroid or mixed culture medium. Scale bar: 10 μm. (**H**) Cytospin images of suspended cells of DMSO group and DZNep-treated groups cultured in erythroid or mixed culture medium. Scale bar: 10 μm. (**I**) Colony-forming ability of cells derived from 0.2 μM DZNep–treated group on day 11; scale bar: 50 μm. (**J**) Proerythroblasts were sorted on day 7 and then treated with DZNep for 72, 96, and 120 hours. Flow cytometry analysis showing the percentage of GPA^–^ cells after treatment. (**K**) Microscopic images of cultured cells including DMSO control and 0.2 μM DZNep–treated groups cultured in erythroid or mixed culture medium; scale bar: 100 μm. (**L**) Cytospin images of suspended cells including DMSO control and 0.2 μM DZNep treated groups cultured in erythroid or mixed culture medium; scale bars: 10 μm. Statistical analysis is from 3 independent experiments, and the bar plot represents mean ± SD of triplicate samples. *P < 0.05, **P < 0.01, ***P < 0.001.

Using an in vitro 3-phase erythroid differentiation system employing CD34^+^ cells from core blood (27, 28), we explored the roles of methionine metabolism in human erythropoiesis by compromising the key enzyme AHCY. AHCY deficiency was induced via doxycycline-inducible shRNA knockdown or DZNep (0.2–0.5 μM) treatment (34, 35) (Figure 1A and Supplemental Figure 1A; supplemental material available online with this article; https://doi.org/10.1172/JCI203565DS1).

During early stage of erythropoiesis, AHCY knockdown (75% efficiency at days 7 and 11, Supplemental Figure 1, B–E) or DZNep treatment significantly reduced cell expansion (Supplemental Figure 1, F and G) due to increased apoptosis (84.7% and 61.2% in knockdown groups, 13.2%–37.8% in DZNep groups versus 8.4% and 5.3% in controls at day 13; Supplemental Figure 1, H–J). Flow cytometry revealed delayed erythroid differentiation with increased BFU-E and decreased CFU-E populations (Supplemental Figure 2, A–C). AHCY deficiency nearly completely blocked the transition to terminal erythropoiesis, as evidenced by the abolished GPA expression from days 7–13 (Supplemental Figure 2, D–F). Morphological analysis confirmed the aberrant differentiation induced by AHCY deficiency. Notably, AHCY deficiency was also found to cause the generation of nonerythroid lineage cells (Supplemental Figure 2G). These results suggest that AHCY deficiency lead to dedifferentiation of erythroid cells or progenitor differentiation block.

During the terminal stage, AHCY knockdown (70% efficiency at day 11; Supplemental Figure 3, A–C) or DZNep treatment significantly impaired the cell expansion. From days 7 to 13, the cell numbers of the control group increased from 1 × 10^6^ to 30 × 10^6^, whereas the cell numbers decreased to 0.5 × 10^6^ of the AHCY-deficient group (Supplemental Figure 3, D and E). Flow cytometry analysis showed a significantly increased apoptosis ratio (40%–57.8% in knockdown, 65%–76% in DZNep groups versus 15.8%–18.3% in controls; Supplemental Figure 3, F–H).

Flow cytometry performed from days 9–13 revealed a significant increase of GPA^–^ erythroblast up to 97.4% in AHCY knock-down group, and up to 92.2% increase in DZNep-treated group, compared with a decrease to 15.2% in controls (Figure 1, B–D, and Supplemental Figure 4, A and B). Notably, these GPA^–^ cells transformed from a suspended state to a semiadherent state. Morphological analysis of the GPA^–^ cells further confirmed the generation of nonerythroid lineage cells (Figure 1E). This finding strongly suggested that AHCY deficiency caused a substantial dedifferentiation during the terminal stage erythropoiesis.

To further identify the GPA^–^ cell generated in response to AHCY deficiency, we cultured DZNep treated cells in erythroid or Pan-myeloid differentiation medium (mix) (36, 37). The results of CCK-8 assay showed that treatment with 0.2 μM DZNep inhibited the cell expansion in erythroid media but sustained it in mixed media until day 20 (Figure 1F). Morphological analysis conducted 8 days later revealed the generation of nonerythroid lineage cells (e.g., macrophage-, neutrophil-, plasma-like) in adherent and suspended fractions (Figure 1, G and H). Further colony forming assays conducted at day 11 showed that DZNep treatment yielded BFU-E, CFU-E, CFU-GM, and CFU-GEMM colonies. Instead, there was no colony formation observed in the control group (Figure 1I). These results indicated that AHCY deficiency actually led to dedifferentiation from erythroblasts to HSPCs.

To exclude the contribution from residual progenitor, GPA^+^ proerythroblasts were isolated using FACS based strategy at day 7. Upon DZNep treatment, the GPA^+^ population decrease from 97% to 1.8% by day 12 (Figure 1J). In contrast, this decrease was not observed in the DMSO control group (Supplemental Figure 4C). Further flow cytometric analysis on the GPA^–^ cell population derived from DZNep-treated erythroblasts at day 11 showed that the vast majority of these cells coexpress CD45, CD33, CD38, CD13, CD11b, CD41, and CD123, well-established markers for human myeloid, megakaryocytic, and early hematopoietic progenitor lineages (Supplemental Figure 4D). A FACS-based method that followed the gating strategy indicated in Supplemental Figure 4E was used to isolate single GPA^+^ erythroblasts. Single cells were sorted into 96-well plates, and only wells containing a single cell (52% of total sorted wells) were selected for subsequent DZNep treatment. Morphology analysis confirmed that DZNep treatment inhibited cell expansion in erythroid media but led to the generation of nonerythroid cells in mixed media by day 30 (Figure 1, K and L, and Supplemental Figure 4F).

Collectively, all these results indicated that AHCY deficiency impairs human erythropoiesis through 2 distinct mechanisms: induction of apoptosis and dedifferentiation of committed erythroblasts into multipotent progenitor-like cells.

### Deficiency of AHCY during terminal erythropoiesis results in reconstitution of the hematopoietic system.

**Figure 2:**
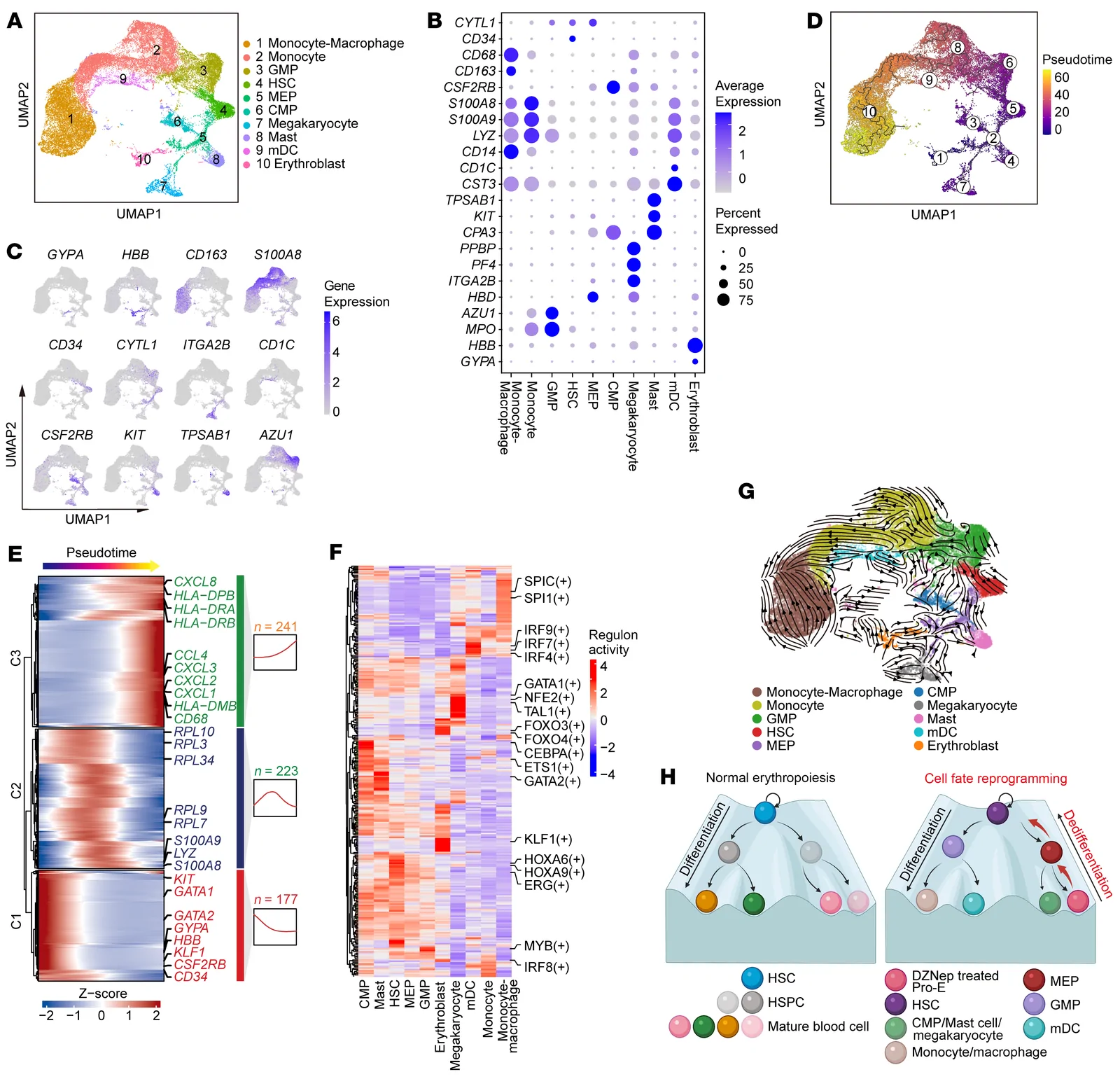
AHCY deficiency leads to cell fate reprogramming of erythroblasts. (**A**) UMAP visualization of all cells displayed with different colors for clusters. (**B**) Dot plots of gene expressions in each subcluster. (**C**) Analysis of mRNA expression in each subcluster. (**D**) The trajectory identified by pseudotime analysis. (**E**) Changes in gene expression with pseudotime trajectories. Three clusters were identified using the k-mean method, and genes with significant changes in expression were labeled. (**F**) Heatmap of regulon activity of TFs in 10 cell clusters. Representative TFs are labeled. (**G**) UMAP plot of the same cells colored by the global RNA synthesis rate (total counts of labeled RNA per cell). (**H**) Schematic illustration of erythroid cell fate reprogramming induced by AHCY deficiency.

To investigate dedifferentiation induced by AHCY deficiency during terminal stage erythropoiesis, we treated the isolated proerythroblasts with 0.2 μM DZNep. After 4 days, cells were analyzed via single-cell RNA sequencing (scRNA-seq) on the 10×Genomics platform. Unsupervised clustering identified 10 cell clusters, annotated by hematopoietic lineage markers (Figure 2, A–C). UMAP revealed DZNep-treated GPA^+^ proerythroblasts dedifferentiated into HSPCs (HSC, MEP, CMP, GMP) and immune cells (macrophages, mDC, monocytes, megakaryocytes, mast cells). Pseudo temporal analysis delineated a precise dedifferentiation trajectory, revealing erythroblasts dedifferentiating into MEP and HSC, then GMP, macrophages, or mDC. Another differentiation trajectory showed MEP differentiating into CMP, megakaryocytes, or mast cells (Figure 2D). DEGs analysis formed 3 clusters: early pseudotime with highly expressed erythroblast (GYPA, HBB, KLF1, GATA1) and stem/progenitor (CD34, GATA2, KIT, CSF2RB) markers, which were then decreased over time; midpseudotime with the elevated expression of monocyte markers (S100A9, S100A18, LYZ); late pseudotime with upregulated expression of MHC-II molecules (HLA-DPB1, HLA-DRA, HLA-DRB1, HLA-DMB) and chemokines (CXCL8, CCL4, CXCL1-3), which were commonly considered to be highly expressed in monocytes, macrophages, and mDC (Figure 2E). SCENIC analysis of gene regulatory networks (GRNs) across cell clusters identified key regulons: GATA1 and KLF1 in erythroblasts, PU.1 in macrophages, and MYB, CEBPA, and ERG in stem/progenitor cells (Figure 2F). Consistent with this, measurement of RNA synthesis rates underscored the presence of the dedifferentiation trajectory (Figure 2G). Bulk RNA-seq of DZNep-treated cells showed upregulated genes in cell fate commitment, myeloid differentiation, and immune response, while downregulated genes were enriched in cell cycle, erythroid differentiation, heme metabolism, and oxygen transport (Supplemental Figure 5, A–G). Collectively, our data indicated that DZNep induced proerythroblast dedifferentiation into multipotent hematopoietic progenitor-like states via reactivation of stemness programs (Figure 2H).

### Transplantation of immunodeficient NCG-X mice with AHCY-deficient erythroid cells lead to the reconstitution of human hematopoietic system.

**Figure 3:**
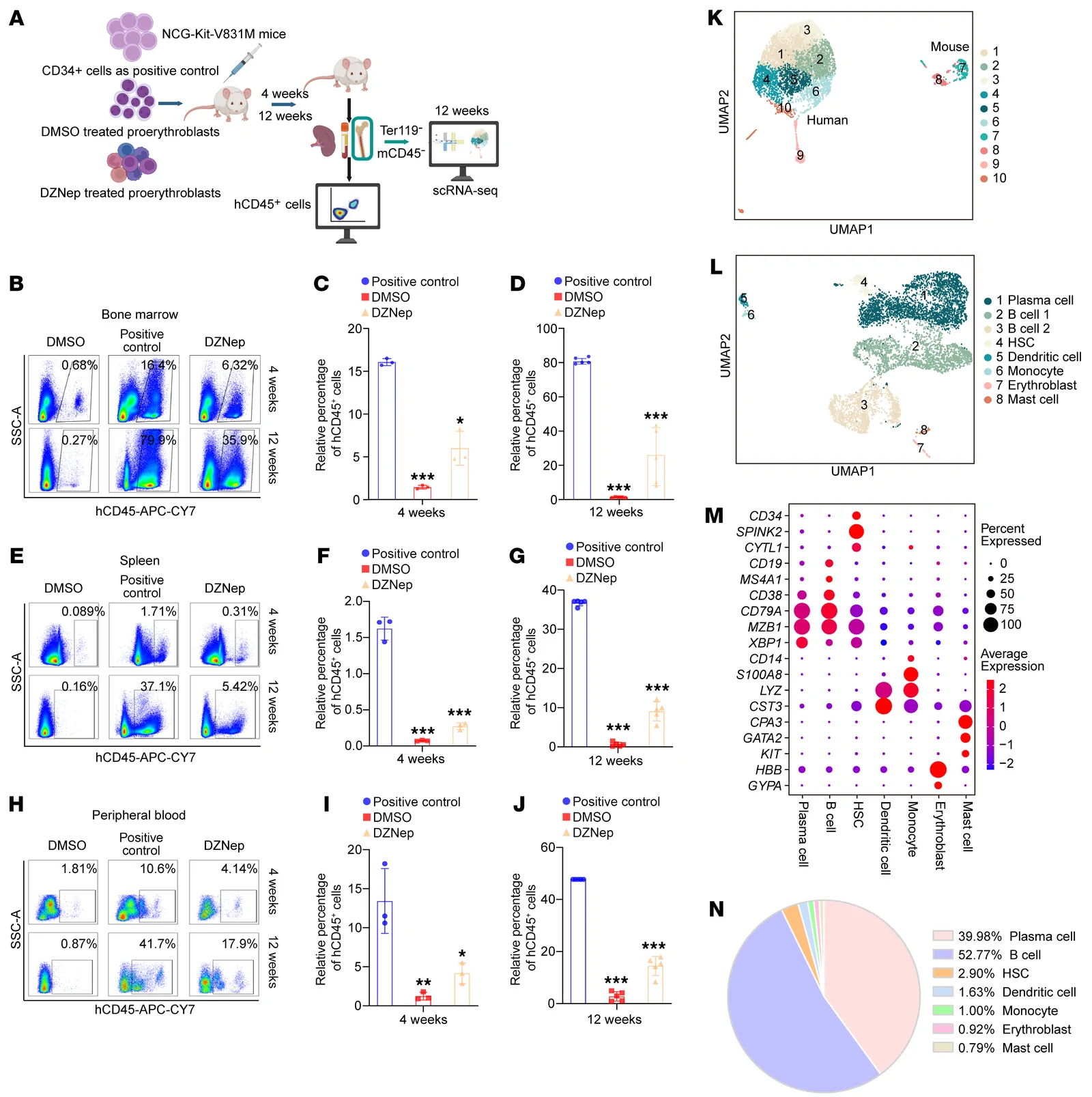
Reconstitution of Human hematopoietic in immunodeficient mice after transplantation of AHCY-deficient erythroid cells. (**A**) Schematic of the experimental protocol for transplanting DZNep-treated proerythroblasts into NCG-X mice and evaluating hematopoietic lineage reconstitution. (**B**) Flow cytometry analysis of BM 4 and 12 weeks after transplantation for determination of human engraftment rates, including DMSO, positive control, and DZNep groups. (**C**) Quantitative analysis of human CD45^+^ cells in BM from 3 independent mice 4 weeks after transplantation. (**D**) Quantitative analysis of human CD45^+^ cells in BM from 5 independent mice 12 weeks after transplantation. (**E**) Flow cytometry analysis of spleen cells 4 and 12 weeks after transplantation for determination of human engraftment rates including DMSO, positive control, and DZNep groups. (**F**) Quantitative analysis of the percentage of human CD45^+^ cells of spleens from 3 independent mice 4 weeks after transplantation. (**G**) Quantitative analysis of the percentage of human CD45^+^ cells of spleens from 5 independent mice 12 weeks after transplantation. (**H**) Flow cytometry analysis of PB 4 and 12 weeks after transplantation for determination of human engraftment rates including DMSO, positive control, and DZNep groups. (**I**) Quantitative analysis of the percentage of human CD45^+^ cells of PB from 3 independent mice 4 weeks after transplantation. (**J**) Quantitative analysis of the percentage of human CD45^+^ cells of PB from 5 independent mice 12 weeks after transplantation. (**K**) UMAP visualization of all human- and mouse-derived cells displayed with different colors for clusters. (**L**) UMAP visualization of all human-derived cells displayed with different colors for clusters after reannotation. (**M**) Dot plots indicate gene expressions in each subcluster. (**N**) Pie chart illustrates the proportional distribution of each cell subpopulation. Statistical analysis is from 3 or 5 independent experiments *P < 0.05, **P < 0.01, ***P < 0.001.

To further validate AHCY deficiency–induced dedifferentiation from erythroblasts to HSC, we performed in vivo transplantation experiments by injecting DZNep-treated proerythroblasts into immunodeficient NCG-X mice, a xenograft model optimized for hematopoietic reconstitution. (38, 39). Proerythroblasts were isolated and treated with DMSO or 0.2 μM DZNep for 4 days, then 2.5 × 10^5^ cells were harvested and injected intravenously into the tail vein of 8–10 week–old mice, with CD34^+^ HSC transplanted mice as positive controls. Time points of 4 and 12 weeks after transplantation were set as the short-term and long-term reconstruction effect assessment, respectively. Bone marrow, spleen, and peripheral blood of the xenografted mice were collected and then subjected to flow cytometry analysis (Figure 3A). As shown in Figure 3, B–D, the human hematopoietic cell chimerism in the bone marrow of mice transplanted with DZNep-treated erythroblasts increased from 6.32% (4 weeks) to 35.9% (12 weeks), compared with 16.4% to 79.9% in the positive controls. In contrast, there was no human chimerism found in the bone morrow of mice transplanted with DMSO-treated erythroblasts. Similar reconstitution was also found in both the spleen (Figure 3, E–G) and peripheral blood (Figure 3, H–J) of the mice transplanted with DZNep-treated erythroblasts and CD34^+^ HSCs.

To further elucidate the reconstitution of the human hematopoietic system in the xenografted mice, bone marrow cells was isolated from the mice transplanted with DZNep-treated erythroblasts and then subjected to scRNA-seq after depleting mouse CD45^+^ and Ter119^+^ cells. UMAP profile showed that human and mouse-derived cells were clustered separately, with clusters 7 and 8, specifically, expressing the mouse genes *Ptma*, *Rps8*, *Malat1,* and *Tmsb4x*, representing mouse-derived cell identity (Figure 3K). We extracted human-derived cells for reclustering, and the UMAP profile clearly identified hematopoietic stem cells, lymphoid lineage cells (B cells and plasma cells), and myeloid lineage cells (monocytes, dendritic cells, and mast cells) (Figure 3, L and M). Consistent with previous studies (39, 40), we observed lymphoid-biased reconstitution of human hematopoiesis in immunodeficient mice transplanted with AHCY-deficient cells (Figure 3N). This lymphoid bias, which was not observed in our ex vivo cytokine-supplemented cultures, likely reflects suboptimal cross-species compatibility of key cytokines and niche factors, a well recognized limitation of humanized mouse xenograft systems. In contrast, ex vivo cultures bypass this species-specific signaling deficiency by providing exogenous cytokines, thereby enabling broader multilineage output. These results thus support AHCY deficiency–driven cell fate reprogramming and the generation of functional hematopoietic stem cells.

Taken together, these results clearly demonstrate that cell fate reprogramming caused by AHCY deficiency is capable to drive the reconstruction of hematopoietic lineages in vivo and in vitro.

### Methionine deprivation impairs human erythropoiesis and leads to the cell fate reprogramming.

As described in the Introduction, methionine metabolism generates SAM, which serves as the primary methyl donor for DNA and histone methyltransferases, thereby directly linking metabolic status to epigenetic regulation. Upon transfer of its methyl group, SAM is converted to S-adenosylhomocysteine (SAH). SAH is a potent feedback inhibitor of methyltransferases, and its removal is exclusively catalyzed by AHCY, the key rate-limiting enzyme in the methionine cycle that hydrolyzes SAH to homocysteine and adenosine. Consequently, the SAH/SAM ratio is a critical indicator of cellular methylation capacity and the overall activity of methionine metabolism. When AHCY is inactivated, SAH cannot be efficiently hydrolyzed, leading to the intracellular accumulation of SAH, SAM and methionine. Thus, to further clarify the impact of AHCY deficiency on methionine metabolism in erythroblasts, we performed metabolomic analyses on erythroblasts treated with 0.2 μM DZNep starting at day 7 and collected at day 11. Principal component analysis (PCA) of the metabolomics data revealed good reproducibility among biological replicates within each group and a clear separation between DZNep-treated cells and DMSO controls (Supplemental Figure 6A). Differential abundance analysis further identified 58 metabolites that were significantly upregulated, 22 that were downregulated, and 101 that remained unchanged upon DZNep treatment (Supplemental Figure 6B). Previous studies have established that disruption of methionine metabolism shifts the SAM/SAH ratio (Supplemental Figure 6C). In line with this, our metabolomics analysis revealed a marked increase in the cellular SAH/SAM ratio after DZNep treatment (Supplemental Figure 6D), as well as markedly elevated intracellular levels of methionine, SAM, and SAH (Supplemental Figure 6, E–G). Collectively, these results demonstrate that AHCY dysfunction indeed disrupts methionine metabolism.

To further dissect the role of methionine cycle flux in terminal erythroblast fate decisions, we performed methionine deprivation experiments. We generated methionine-free medium (Met Free) and compared cell growth and differentiation with control amino acid complete medium (AA Complete). Methionine depletion strongly reduced cell proliferation (Supplemental Figure 7A). Flow cytometry analysis showed a significantly increased apoptosis rate (67.1%–73.8% in Met-free group versus 10.06%–35.5% in controls; Supplemental Figure 7, B and C). From day 9 to day 13, the proportion of GPA^–^ erythroblasts increased dramatically to 82.2% in the Met-free group, whereas it decreased to 15.3% in the control group (Supplemental Figure 7, D and E). As a canonical myeloid marker, CD33 positivity increased from 5.87% to 36.9% upon methionine deprivation (Supplemental Figure 7, F and G). Morphological analysis of GPA^–^ cells further confirmed the generation of nonerythroid lineage cells (Supplemental Figure 7H). These findings strongly suggest that methionine deprivation induces cell fate reprogramming during terminal erythropoiesis, phenocopying AHCY deficiency.

### Aberrant H3K4me3 modification caused by AHCY deficiency leads to the cell fate reprogramming.

Given that methionine metabolism provides the methyl donor SAM for DNA, RNA, and histone methylation, we further investigated whether AHCY dysfunction broadly impairs DNA and RNA methylation. Dot blot assays revealed that levels of 5-methylcytosine (5mC) and N6-methyladenosine (m^6^A) were significantly reduced in DZNep-treated proerythroblasts compared with DMSO-treated controls (Supplemental Figure 8, A–D). These results indicate that AHCY deficiency broadly impairs DNA and RNA methylation, in addition to the histone methylation modifications described above.

**Figure 4:**
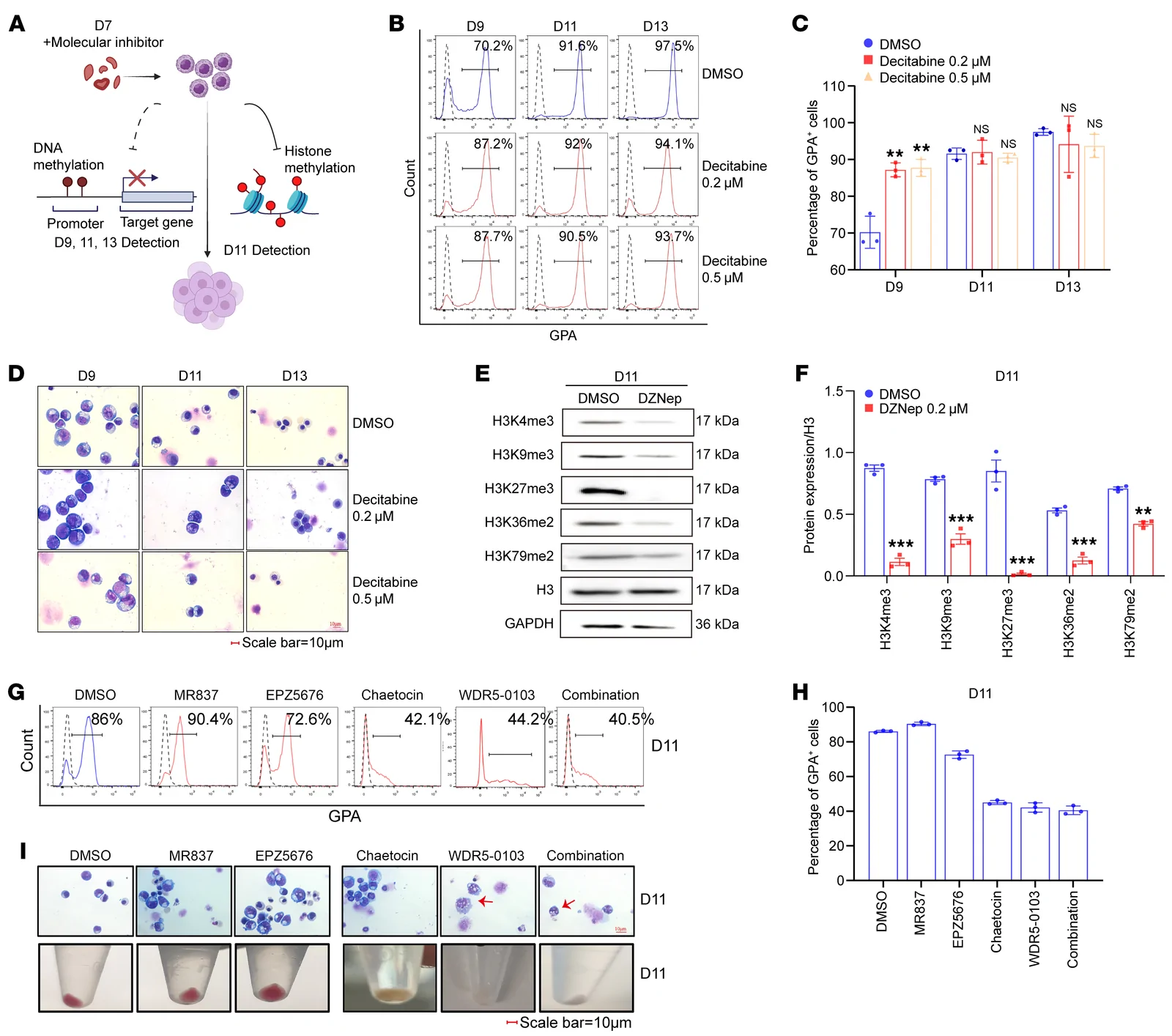
Reduction of H3K4me3 leads to cell fate reprogramming of erythroblast. (**A**) Schematic of experimental outline for the treatment with specific inhibitors of DNA methylation and histone methylations. (**B**) Flow cytometry analysis showing the percentage of GPA^–^ cells on days 9, 11, and 13. (**C**) Quantitative analysis of GPA^–^ cells from 3 independent experiments. (**D**) Representative cytospin images of DMSO and Decitabine (0.2 μM, 0.5 μM) groups on days 9, 11, and 13. Scale bar: 10 μm. (**E**) Representative Western blot images showing significantly downregulated histone methylations after treatment with 0.2 μM DZNep. (**F**) Quantitative analysis showing the levels of the 5 histone methylations from 3 independent experiments. (**G**) Flow cytometry analysis showing the percentage of GPA^–^ cells after treatment with various specific inhibitors for histone methylation. (**H**) Quantitative analysis of GPA^–^ cells from 3 independent experiments. (**I**) Representative cytospin images and cell pellet color of histone methylation inhibitor–treated group, morphological identified nonerythroid lineage cells are indicated by red arrowhead. Scale bar: 10 μm. Statistical analysis is from 3 independent experiments, and the bar plot represents mean ± SD of triplicate samples. **P < 0.01, ***P < 0.001.

To investigate whether AHCY deficiency–induced lineage infidelity in terminal erythropoiesis is mediated by aberrant DNA methylation, we treated proerythroblasts with decitabine (0.2/0.5 μM) to inhibit DNA methylation (41, 42). At day 7, cells were treated with decitabine, and the proliferation and apoptosis were determined every 2 days until day 13. The results showed that decitabine (0.2/0.5 μM) treatment significantly inhibited the cell expansion, with cell numbers dramatically decreased to 16 × 10^6^ and 5 × 10^6^ at day 13, respectively (Supplemental Figure 9A). Decitabine treatment also led to a significant increase of apoptotic cell ratio to 26.3% and 39%, respectively (Supplemental Figure 9, B and C). However, flow cytometry analysis showed that decitabine treatment did not affect GPA^+^ erythroblast ration (Figure 4, B and C). The morphological analysis also indicated that decitabine did not affect the differentiation of erythroblast during terminal stage (Figure 4D). Thus, these findings suggested that, although DNA methylation plays critical roles in the maintaining of cell expansion, it shows no effect on the cell fate determination.

We therefore checked the influence of AHCY deficiency on histone methylation. Cells were treated with 0.2 μM DZNep at day 7 and were collected at day 11 and then subjected to Western blot assay to detect the level of key histone methylations. The results showed that H3K4me3, H3K9me3, H3K27me3, H3K36me2, and H3K79me2 were substantially reduced in respond to AHCY deficiency (Figure 4, E and F). Since our recent work has shown that reduction of H3K27me3 during terminal stage did not affect cell differentiation during terminal stage (43), we therefore intend to identify the key player from the other 4 histone modifications that is responsible for the cell fate reprogramming.

We treated the erythroblasts at day 7 with the specific inhibitors of these 4 histone methylations (Supplemental Table 3), individually or in combination, and then check the cell expansion and cell differentiation 4 days after (Figure 4A). Treatment with EPZ5676 (inhibitor of H3K79me2), Chaetocin (inhibitor of H3K9me3), or WDR5-0103 (inhibitor of H3K4me3) individually or in combination all significantly inhibited the cell expansion by inducing apoptosis, whereas treating with MR837 (inhibitor of H3K36me2) showed no effect (Supplemental Figure 10, A–C). WDR5-0103, Chaetocin, and combined treatments reduced GPA^+^ cells to approximately 40% (Figure 4, G and H). Notably, cell pellets from WDR5-0103 and combined inhibitor groups were colorless, indicating a severe impairment of erythroid differentiation, while Chaetocin-treated pellets appeared light red, suggesting partial differentiation disruption. It was interesting to note that the cell pellets collected from WDR5-0103 and combined inhibitor treatment groups were colorless, the cell pellets from the Chaetocin-treated group appears to be light red, while the other groups appeared deep red. This finding indicated that the cell differentiation of Chaetocin and WDR5-0103–treated group were significantly impaired (Figure 4I). Cytospin assays identified specific generation of nonerythroid lineage cells (arrowheads) in WDR5-0103 and combination groups (Supplemental Figure 10D). The dose response was validated in parallel by flow cytometry (Supplemental Figure 10E) and by observing the loss of characteristic red pellet color (Supplemental Figure 10F) across a WDR5-0103 gradient (Supplemental Figure 10, E and F). Taken together, all these results strongly support a primary role for H3K4me3 inhibition in the cell fate reprogramming of AHCY-deficient erythroblasts; however, we cannot categorically exclude a possible contribution of H3K9me3.

### Integrated analysis reveals the transcriptional reprogramming induced by altered H3K4me3 landscape caused by AHCY deficiency.

**Figure 5:**
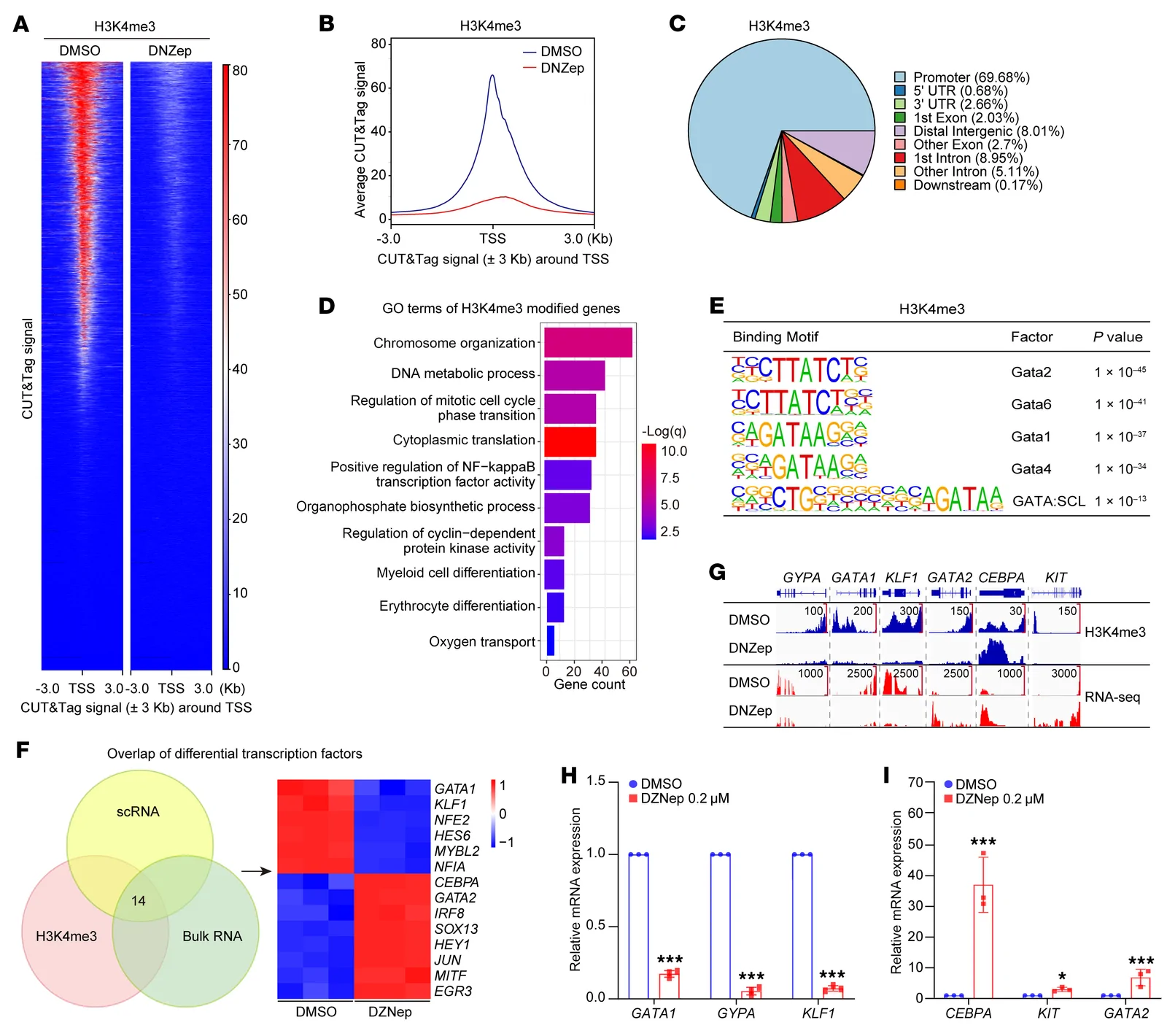
Reshaped H3K4me3 landscape causes remodeling expression pattern of lineage-specific transcription factors. (**A**) The heat maps showing the CUT&Tag signals of DMSO control (left) and 0.2 μM DZNep (right) around TSS. (**B**) Representative peaks chart image showing the CUT&Tag signals of DMSO control (blue) and 0.2 μM DZNep (red) around TSS. (**C**) The pie chart showing the distribution of different peaks relative to gene features. (**D**) GO analysis showing the functional classification of downregulated DEGs that are regulated by H3K4me3 following treatment with 0.2 μM DZNep. (**E**) Top TF motifs enriched in H3K4me3 CUT&Tag peaks linked to promoters. (**F**) Venn plot and heat map of CUT&Tag, scRNA-seq, and RNA-seq showing TF gene expression enriched for H3K4me3. (**G**) Histone modification and gene expression levels of *GYPA*, *GATA1*, *KLF1*, *GATA2*, *CEBPA*, and *KIT* loci in DMSO control and 0.2 μM DZNep. (**H**) Quantitative analysis of the relative mRNA expression level of *GATA1*, *GYPA*, and *KLF1* from 3 independent experiments. (**I**) Quantitative analysis of the relative mRNA expression level of *CEBPA*, *KIT*, and *GATA2* from 3 independent experiments. Statistical analysis is from 3 independent experiments, and the bar plot represents mean ± SD of triplicate samples. *P < 0.05, ***P < 0.001.

To further elucidate the mechanism by which AHCY deficiency causes cell fate reprogramming through reshaping H3K4me3 landscape, we conducted integrated analysis of CUT&TAG and RNA-seq on erythroblasts that were treated with 0.2 μM DZNep at day 7, and collected at day 11. CUT&Tag sequencing was performed to explore the genome-wide distribution of H3K4me3. PCA and Spearman correlation analysis confirmed high concordance among 3 biological replicates of DZNep-treated and control groups (Supplemental Figure 11, A and B). CUT&Tag analysis revealed 12,072 lost peaks, 10,538 shared peaks, and 1,350 gained H3K4me3 peaks after treatment with DZNep, with significantly reduced signals in the vicinity of transcription start sites within a 3 Kb region (Figure 5, A and B, and Supplemental Figure 11C). Genomic annotation showed that the lost peaks were enriched in promoter regions (69.98%, Figure 5C). GO analysis showed that DEGs associated with the lost H3K4me3 peaks were linked to chromosome organization, myeloid cell/erythroid differentiation, and oxygen transport (Figure 5D). HOMER analysis showed that H3K4me3 peaks enriched at erythroid-specific TF binding sites (GATA1, KLF1, TAL1, GATA2) in controls (Figure 5E), whereas there was a shift to nonerythroid lineage-specific TF (FIL1, ETS, ERG) binding motifs after DZNep treatment (Supplemental Figure 11D). Further Integrated analysis of scRNA-seq, bulk RNA-seq, and H3K4me3 peak intensities identified 14 TFs (Figure 5F, left), with erythroid TFs (GATA1, KLF1) downregulated and stem/progenitor TFs (CEBPA, GATA2) upregulated after DZNep treatment (Figure 5F, right). RNA-seq and CUT&Tag-seq signal tracks, annotated to gene loci, show changes in the intensity of H3K4me3 peaks, along with alterations in the expression levels of GYPA, GATA1, KLF1, CEBPA, and KIT (Figure 5G). These results were also validated by qRT-PCR (Figure 5, H and I). In summary, these results highlight that H3K4me3 critically regulates lineage-specific TF expression during terminal erythropoiesis. AHCY deficiency reshapes the H3K4me3 landscape, driving transcriptional reprogramming of these TFs and ultimately causing cell fate reprogramming.

### Reduction of H3K4me3 in erythroblasts are tightly associated with AML pathogenesis.

Given the established roles of H3K4me3 dysregulation in leukemia (44–47) and the frequent cooccurrence of erythropoiesis defects in myeloid malignancies (48–51), we hypothesized that AHCY deficiency in erythroblasts remodels the H3K4me3 landscape, potentially contributing to myeloid neoplasm development and leukemia-associated anemia.

**Figure 6:**
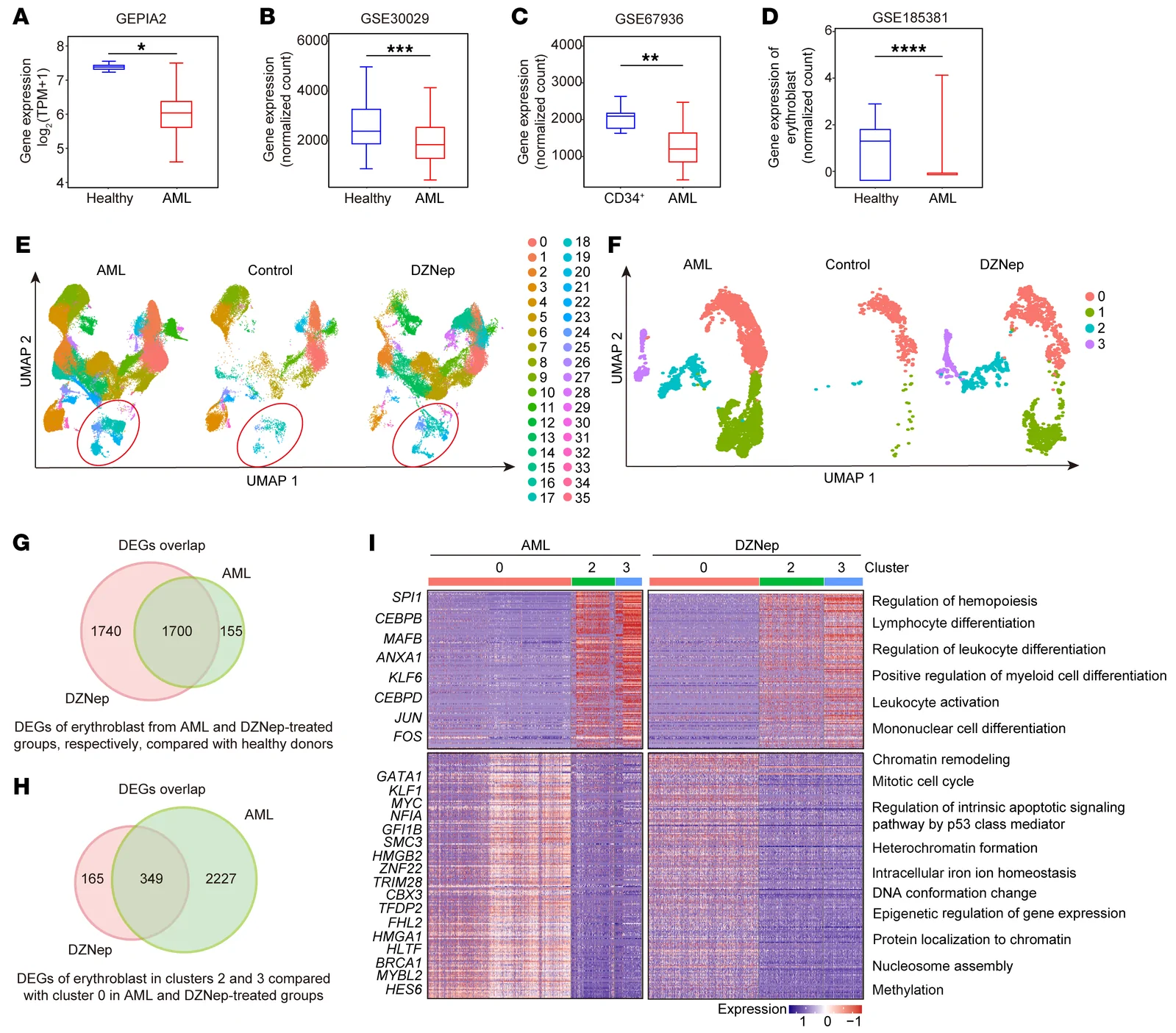
Transitional state cells derived from erythroblasts with reduced expression of *AHCY* exist in patients with AML. (**A**–**C**) Box plot of *AHCY* expression level in AML and healthy controls (**A** (GEPIA2), **B** (GSE30029), **C** (GSE67936)). (**D**) Box plot of *AHCY* expression level in erythroblasts from patients with AML and healthy controls (GSE185381). (**E**) UMAP visualization of all cells displayed with different colors for clusters. (**F**) UMAP visualization of erythroblasts displayed with different colors for clusters. (**G**) Venn diagram showing the overlap of AML and DZNep-treated erythroblasts with DEGs from healthy controls, respectively. (**H**) Venn diagram showing the overlap of clusters 2 and 3 compared with cluster 0 in AML erythroblasts with DEGs from DZNep-treated samples. (**I**) Heat map and GO enrichment analysis of terms enriched for 349 high-confidence DEGs of erythroblast in clusters 2 and 3 compared with cluster 0 in AML and DZNep-treated groups.

We examined AHCY expression in acute myeloid leukemia (AML) patients using public gene expression microarrays (GSE30029 and GSE67936) and RNA-seq datasets (GEPIA2), revealing significantly lower AHCY expression in patients with AML (Figure 6, A–C). Further analysis of erythroblasts from 31 patients with AML and 5 healthy individuals, based on a single-cell sequencing dataset (GSE185381) (52), revealed that AHCY was downregulated in the erythroblasts of patients with AML (Figure 6D). To compare AHCY-low erythroblasts in AML with methionine metabolism–deficient erythroblasts, we integrated scRNA-seq data from DZNep-treated erythroid cells with data from patients with AML (Figure 6E). Unsupervised clustering identified 35 clusters, with clusters 17, 21, and 29 annotated as erythroblasts based on CD34^–^CD36^+^IL3RA^–^SLC4A1^+^GYPA^+^ expression (Supplemental Figure 12A). Although numerous DEGs were identified in AML (*n* = 1,855) and DZNep-treated (*n* = 3,440) erythroblasts compared with healthy controls, only 34 DEGs differed between AML and DZNep groups (Supplemental Figure 12B). A large proportion of DEGs (*n* = 1,700) were shared between AHCY-low erythroblasts in AML and DZNep-treated erythroblasts relative to controls (Figure 6G), indicating transcriptional similarity between them.

Subclustering of erythroblasts identified 4 clusters. Clusters 1–3 were predominantly composed of AML and DZNep-treated cells, whereas cluster 0 consisted mainly of healthy controls (Figure 6F). The transcriptional similarity between AML and DZNep-treated erythroblasts, coupled with their shared AHCY deficiency, led us to classify clusters 1, 2, and 3 as abnormal erythroblasts. These aberrant erythroblasts coexpressed erythroid markers (*TFRC*, *GYPA*, *HBB*) and nonerythroid lineages markers (e.g., *CD68*, *S100A8*, *MPO*) (Supplemental Figure 12C). These aberrant erythroblasts exhibited downregulation of erythroid TFs (*GATA1, TAL1, KLF1*) and upregulation of nonerythroid TFs (e.g., *SPI1, IRF7, KLF2*) (Supplemental Figure 12D).

A core set of 349 high-confidence DEGs, including key hematopoietic TFs such as *SPI1*, *CEBPB*, *MYC*, *GFI1B*, *GATA1*, and *KLF1*, that were identified from these clusters was enriched for pathways in chromatin remodeling, myeloid differentiation, and p53 signaling (Figure 6, H and I).

We identified 349 high-confidence DEGs compared with cluster 0 in AML and DZNep groups (Figure 6H), including key hematopoietic TFs such as *SPI1*, *CEBPB*, *MYC*, *GFI1B*, *GATA1*, and *KLF1* (Figure 6I). Overexpression of *SPI1* (encoding PU.1) is known to drive oncogenic transformation in proerythroblasts. Gene ontology analysis revealed enrichment in chromatin remodeling, regulation of hematopoiesis, leukocyte differentiation, myeloid differentiation, cell cycle, and p53-mediated apoptotic signaling (Figure 6I). Notably, TP53 mutations are a hallmark of acute erythroleukemia, further underscoring the relevance of these pathways.

**Figure 7:**
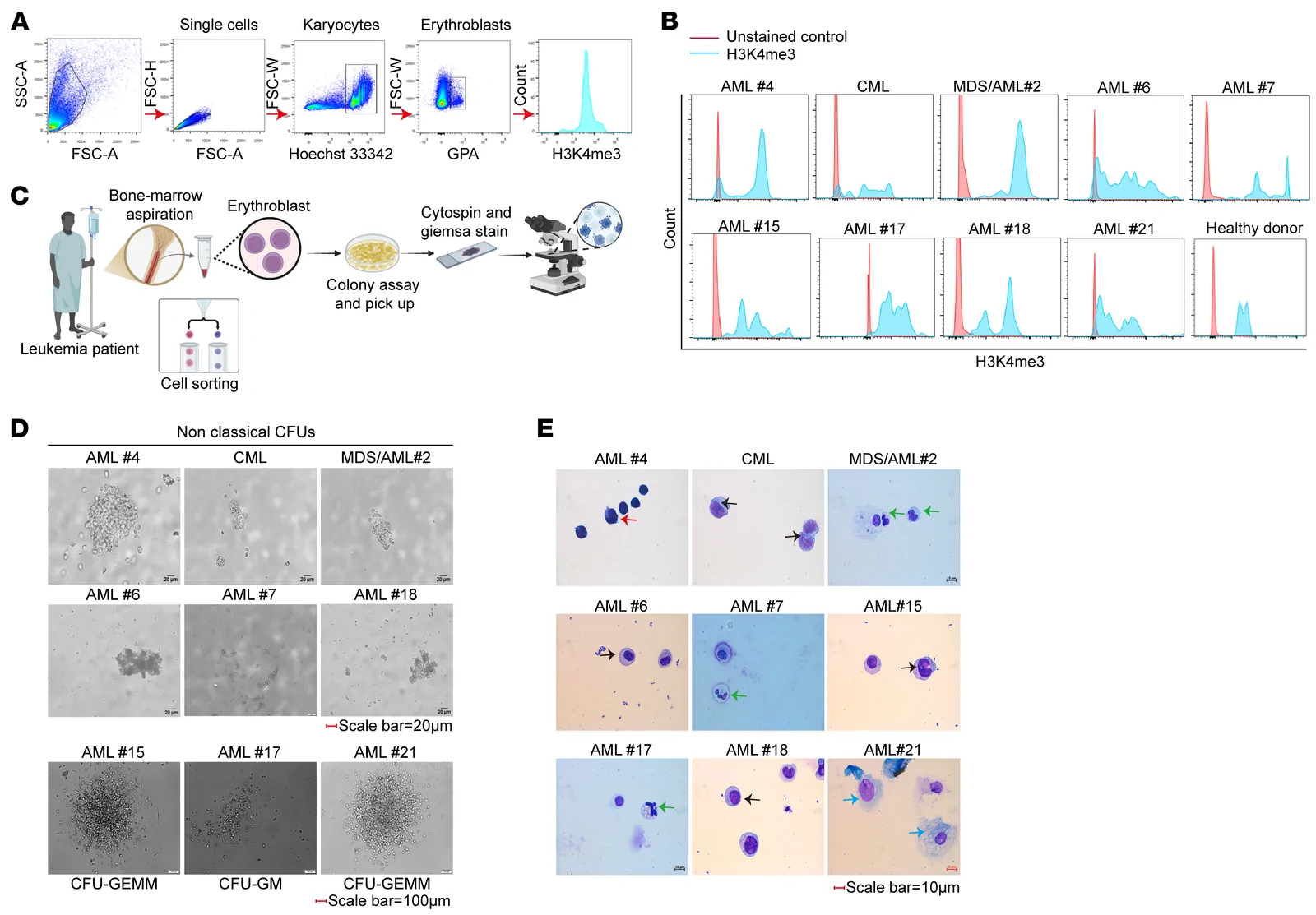
Reduced H3K4me3 level of erythroblasts leads to the generation of progenitor-like cells. (**A**) Gating strategy for the flow cytometry analysis of the level of H3K4me3 in erythroblasts of bone marrow from leukemia patients. (**B**) Flow cytometry analysis showing the heterogeneity pattern of existence of H3K4me3 in erythroblasts from 1 healthy donor and 9 patients with myeloid leukemia. (**C**) Schematic of experimental outline for the colony forming assay of erythroblast from the 9 patients with myeloid leukemia. (**D**) Morphological observation of the colonies forming by the erythroblasts from the 9 patients with myeloid leukemia. Scale bar: 20 μm and 100 μm. (**E**) Smear of cells aspirated from a colony stained with Giemsa. Black arrowhead, monocyte; granulocyte, green arrows; erythroid and megakaryocyte progenitors, red arrows, macrophage, yellow arrows. Scale bar: 10 μm.

We identified 349 high-confidence DEGs in clusters 2 and 3 in AML and DZNep-treated groups (Figure 6H), including haematopoietic TFs, such as *SPI1, CEBPB, MYC, GFI1B, GATA1,* and *KLF1* (Figure 6I). GO analysis revealed their enrichment in chromatin remodeling, regulation of hemopoiesis, lymphocyte differentiation, myeloid differentiation, cell cycle, p53-mediated apoptosis (Figure 6I), pathways are well established in leukemogenesis (53, 54). We therefore defined these aberrant erythroblasts emerged in patients with AML as “transitional state cells”, likely originating from *AHCY-*deficient erythroblasts. We hypothesized that AHCY deficiency induces H3K4me3 landscape remodeling in erythroblasts, which, in turn, contributes to the pathogenesis of AML through the emergence of transitional state cells. Flow cytometry analysis of bone marrow samples from 32 patients with myeloid neoplasms (MDS, CML, AML) (Supplemental Table 4) showed heterogeneous or markedly reduced H3K4me3 levels in GPA^+^ erythroblasts in nearly half of the samples (15 of 32) (Figure 7, A and B), predominantly in AML (13 of 15) (Supplemental Figure 13, A–C). In contrast with the consistently high and homogeneous H3K4me3 levels in healthy donor erythroblasts, patient erythroblasts exhibit marked heterogeneity in H3K4me3, with a subset of cells retaining relatively high levels while another subset shows substantially reduced levels. Importantly, this pattern aligns closely with our morphological observations and our prior integrated single-cell transcriptomic analyses, which identified a distinct “transitional state” erythroid population marked by loss of lineage identity and gain of plasticity. We infer that the low-H3K4me3 erythroblasts likely represent this transitional population.

Given the potential role of H3K4me3 reduction in cell fate reprogramming, we wondered whether erythroblasts with low H3K4me3 could give rise to aberrant HSPCs. GPA^+^ erythroblasts from 9 patients with AML (Supplemental Table 4, red) were isolated by FACS and cultured in semisolid medium (Methocult H4434, STEMCELL) with additional cytokines including Flt3 Ligand (50 ng/mL), G-CSF (10 ng/mL), M-CSF (100 ng/mL), and GM-CSF (5 ng/mL) (Figure 7C). After 14 days, colony-forming assays revealed multiple colony types, including CFU-GEMM, CFU-GM, and nonclassical colonies (Figure 7D). Cytospin analysis confirmed the presence of monocyte-, macrophage-, and granulocyte-like cells within these colonies (Figure 7E, arrowheads). Notably, these cell lineages generated from erythroblasts were similar to abnormal proliferative linearity of the patients, which was confirmed by clinical diagnosis.

Collectively, these data support our hypothesis that the reduction of H3K4me3 in erythroblasts reshapes the cell fate decision and generates progenitor-like cells, contributing to myeloid leukemia–associated anemia.

## Discussion

Here, we uncovered the critical role of methionine metabolism in human erythropoiesis, where it sustains cell expansion and shapes cell fate decisions. Disruption of methionine metabolism by AHCY deficiency severely impaired erythropoiesis through 2 mechanisms: causing cell apoptosis by blocking DNA methylation and leading to cell fate reprogramming by reshaping H3K4me3 landscape. Reduced H3K4me3 levels in erythroblasts are strongly linked to the pathogenesis of myeloid neoplasm–associated anemia.

Beyond the critical roles of DNA and histone methylation, emerging evidence highlights pivotal functions for nonhistone and RNA methylation in erythropoiesis, both directly coupled to methionine metabolism. PPRMT1-mediated p38α methylation promotes erythroid differentiation (55, 56). EZH2-dependent HSP70 methylation is essential for nuclear condensation and enucleation during terminal erythropoiesis (43). Additionally, the m^6^A modification machinery (METTL3/14/WTAP) maintains GPA expression during erythroid lineage commitment, a role underscored by genome-wide CRISPR screens (57). These methyltransferase-dependent links underscore a broad metabolic-epigenetic regulatory network (58, 59).

Disruption of methionine metabolism through AHCY inhibition by DZNep facilitates pluripotent reprogramming through demethylation of DNA and histone H3K9, which activates *OCT4* expression, establishing DZNep as a key component in chemical reprogramming protocols to drive intermediate-state cells toward pluripotency (12, 14, 60). However, during human erythropoiesis, DZNep-mediated disruption of methionine metabolism produces fundamentally distinct outcomes. Our findings reveal that DZNep treatment does not alter pluripotent gene expression, despite profoundly disrupting DNA and histone methylation. Instead, DZNep induces extensive remodeling of H3K4me3 landscapes, substantially upregulating nonerythroid lineage-specific TFs while downregulating erythroid-specific ones. This distinct mechanism of methionine metabolism disruption directs a unique erythroid lineage-specific cell fate trajectory, promoting dedifferentiation into hematopoietic lineage cells rather than pluripotent cells, highlighting context-dependent reprogramming outcomes.

Another intriguing finding from our study is the identification of intermediate-state erythroblast-like cell subpopulations in patients with AML and DZNep-treated cells. While these subpopulations express erythroid-specific markers, such as *GYPA*, *HBB*, and *TFRC*, they also exhibit expression of nonerythroid lineage-specific genes, including myeloid markers (e.g., *CD14*, *CD68*, *CD163*) and lymphoid markers (e.g., *SPI1*, *IRF7*, *IRF8*). This mixed-lineage expression pattern suggests that these aberrant erythroblast-like cells are transitioning from an erythroid identity toward undetermined cell fates. Analogous to the intermediate plastic cells that emerge during the early phases of small molecule–induced cell fate reprogramming, the appearance of these erythroblast-like cells marks the initial stage of erythroid lineage reprogramming. We thus designate them as transitional-state cells. The subsequent trajectory of these transitional-state cells is likely shaped by the culture conditions or hematopoietic microenvironment, accounting for the diverse cell types observed across our in vitro assays, immunodeficient mouse xenografts, and patient-derived colony-forming assays.

H3K4me3 serves as a critical epigenetic activation mark at gene promoters, associating with open chromatin and transcriptional activity. Chromatin dynamics analysis using indexing-first chromatin immunoprecipitation (iChIP) during hematopoietic differentiation revealed a 30%–40% reduction in genome-wide H3K4me3 levels as hematopoietic stem cells (HSCs) differentiate to mature blood cells (61). Knockdown of the H3K4me3 demethylase Jarid1b (KDM5B) elevates H3K4me3 at critical HSC genes, thereby blocking differentiation (62). An aberrant H3K4me3 landscape has been demonstrated to be tightly associated with concurrence of various types of leukemia. For instance, mutations in PHD fingers that disrupt H3K4me3 binding abolish leukemic transformation. NUP98-PHD fusion, which was identified in leukemias, has been shown to prevent the differentiation-associated removal of H3K4me3 at many loci that encode lineage-specific TFs. The fusion also enforced the active gene transcription of these TFs in HSPCs, thus severely impairing hematopoiesis (63). In chronic lymphocytic leukemia (CLL), KDM1A is identified as an oncogenic transcriptional repressor that alters H3K4me3 patterns with pronounced effects on defined cell death and motility pathways. Pharmacologic KDM1A inhibition altered H3K4/9 target methylation and revealed marked anti–B cell leukemic synergisms. H3K4-specific demethylase KDM5C also has been identified as a tumor suppressive epigenetic regulator in AML. KDM5C deficiency induced globally increased H3K4me3 levels associated with upregulation of bivalently marked immature genes (64). These studies demonstrate that, beyond disruptions in methionine metabolism, multiple factors can induce aberrant H3K4me3 landscapes, contributing to leukemogenesis. Further investigation is warranted to elucidate the processes and molecular mechanisms underlying erythroid cell fate reprogramming associated with H3K4me3 landscape alterations and driven by factors other than methionine cycle impairment. More investigation is also required to devise effective strategies for restoring the appropriate lineage-specific H3K4me3 landscape using in vivo and in vitro models.

## Methods

### Sex as a biological variable.

All animal studies included equal representations of male and female mice. Both male and female patients were involved for sample collection.

### Culture of CD34^+^ cells and shRNA mediated knockdown.

Primary human CD34^+^ cells from cord blood were isolated from mononuclear cells (MNC) obtained by human peripheral blood lymphocyte isolation fluid and followed by positive selection using CD34^+^ magnetic beads system (Miltenyi Biotechnology, Germany) according to the manufacturer’s protocol. The detailed culture medium composition, the culture protocol, preparation of lentivirus, and transduction in CD34^+^ cells have been described previously (26, 27, 28). The sequences of shRNA oligos are listed in Supplemental Table 2.

### Drug treatment.

The erythroid cells were incubated in the presence of dimethyl sulfoxide (Sigma-Aldrich) or various types of drugs. AHCY inhibitor DZNep and DNA methylation inhibitor Decitabine were purchased from MCE, (MCE, NJ), Histone methylation inhibitor Tazemetostat, Pinometostat, Chaetocin (S8068) were purchased from Selleck, MR837 and WDR5-0103 were purchased from MCE. The detailed information for the drugs was described in the Supplemental Materials and Methods.

### Antibodies.

Mouse monoclonal antibody against human band 3 was generated in our laboratory and labeled with FITC or APC as described previously (65). Other antibodies and reagents used for flow cytometry, western blot and immunofluorescence analyses are listed in Supplemental Table 1.

### CUT&Tag.

Cells were collected and sequentially incubated with ConA Beads, primary antibody and Hyperactive PG-TN5/PA-TN5 Transposon and then fragmented. The fragmented DNA was extracted from the samples and amplified by PCR. CUT&Tag libraries were constructed using HyperactiveTM In-Situ CUT&Tag Library Prep Kit (TD901, Vazyme, China) and sequenced on an Illumina NovaSeq platform. Details of data generation and analyses are described in the Supplemental Materials and Methods.

### RNA-seq and bioinformatics analysis.

RNA was extracted and used as cDNA input, cDNA library was prepared by Illumina Truseq DNA library kit, and sequenced on Illumina NovaSeq 6000. Details of data generation and analyses are described in the Supplemental Materials and Methods.

### scRNA-seq.

Cells were collected with or without drug treatment and used for single-cell suspension preparation and quality inspection. According to the library construction process officially recommended by 10×Genomics, library construction and on-board sequencing were performed. Details of data generation and analyses are described in the Supplemental Materials and Methods.

### Transplantation and engraftment analysis.

NCG-X mice were obtained from GemPharmatech in China and the related experiments were approved by the Institutional Animal Care and Use Committee of Zhengzhou University. Adherent and suspended cells were obtained after treated with or without 0.2 μM DZNep. Non-irradiated NCG-X female mice (3–4 weeks age) were subjected to tail intravenous injection with 0.675 million cells (resuspended in 300 μL DPBS). Equal numbers of CD34^+^ HSPCs were injected into NCG-X mice, which served as controls. We performed flow cytometry analysis of BM cells for monitoring of human engraftment efficiency 4 and 12 weeks post transplantation. A DZNep group mouse bone marrow sample removed mouse CD45^+^ and Ter119^+^ cells was utilized to conduct scRNA-seq and bioinformatics analysis.

### Cell metabolomics analysis.

Cells were cultured, washed with ice-cold PBS, quenched with liquid nitrogen, and extracted using a pre-chilled mixture of methanol/acetonitrile/water (2:2:1, v/v/v) containing an internal standard (2-chloro-L-phenylalanine, 10 μg/mL). After 3 freeze–thaw cycles and sonication, the extract was centrifuged at 14,000 × g for 15 min at 4°C, and the supernatant was dried under nitrogen, reconstituted in 50% acetonitrile/water, and analyzed by LC-MS/MS on a C18 column using a gradient of water and acetonitrile (both with 0.1% formic acid) at 0.3 mL/min over 15 min. Mass spectrometry was performed on an Orbitrap instrument in positive and negative ion modes with full scan range m/z 70–1050. Raw data were processed using Compound Discoverer, metabolites identified by matching against HMDB and METLIN, and data normalized by total ion count. Statistical analysis including PCA and OPLS-DA was carried out with SIMCA-P, and metabolites with VIP > 1 and *P* < 0.05 (Student’s t-test) were considered significantly different.

### Statistics.

All experiments were replicated at least 3 times. Statistical analysis was performed with SPSS 20.0 software. Data was presented as a mean value with 95% confidence interval (CI). *P* < 0.05 were considered to be statistically significant.

### Study approval.

This study is approved by the Ethics Committee of Zhengzhou University (Approval No.:ZZUIRB2024-77). BM aspiration samples were collected from patients with newly diagnosed or currently untreated CML, AML and MDS patients undergoing routine diagnostic evaluation after written informed consent. BM samples of healthy individuals without known hematologic disease were obtained from femoral heads after hip replacement surgery after written informed consent. BM aspiration samples were transported directly after collection procedures to our laboratory under sterile conditions. The investigations were conducted in accordance with the Declaration of Helsinki. Patient characteristics are shown in Supplemental Table 4. The BM mononuclear cells (MNCs) were isolated by density gradient centrifugation through ficoll (density: 1.077 g/L). NCG-Kit-V831M mice were purchased from GemPharmatech Co., Ltd (Nanjing, China). All mice were kept under specific pathogen-free conditions, and all procedures were performed according to protocols approved by the Institutional Animal Care and Use Committee of GemPharmatech.

### Data availability.

The RNA-seq, CUT&Tag and scRNA-seq datasets for erythroid cells induced by human umbilical cord blood-derived CD34^+^ hematopoietic stem cells generated during the current study are available at the GEO under the accession codes GSE270051 (bulk RNA-seq), GSE270052 (CUT&Tag) and GSE270053 (scRNA-seq). The scRNA-seq dataset for mouse bone marrow mononuclear cells depleted of mouse CD45^+^ and Ter119^+^ cells generated in this study are available at the GEO under the accession codes GSE270054. Values for all data points in graphs are reported in the Supporting Data Values file. The metabolomics datasets are available at MetaboLights under the accession code MTBLS10469. There are no restrictions on data availability.

## Author contributions

LC, XA, and TW designed the overall project, analyzed the results, and prepared the manuscript, with input from all coauthors. LS performed the experiments with assistance from XW, YC, and YS; HZ performed the integrated bioinformatics analysis with assistance from YH and JL; ML performed the analysis of the patient sample–based investigation with assistance from QL, SW, YW, and YS. Author order among co–first authors was determined by mutual agreement.

## Conflict of interest

The authors have declared that no conflict of interest exists.

## Funding support

- National Natural Science Foundation of China (82470118, 82170116 and 81870094, to LC).
- National Natural Science Foundation of China (82400140, to YC).

## Supplementary Material

Supplemental data

Unedited blot and gel images

Supporting data values

## Version 1 07/14/2026

In-Press Preview

## Version 2 09/01/2026

Electronic publication
