# Supplementary material for: Disruption of methionine metabolism drives erythroid cell fate reprogramming by remodeling the H3K4me3 landscape
Source: J Clin Invest. 2026 Jul 14;136(17):e203565. doi: 10.1172/JCI203565 (PMC13528939; doi:10.1172/JCI203565)
Supplement: Supplementary file 1 [file jci-136-203565-s098.pdf]

Supplemental Figures

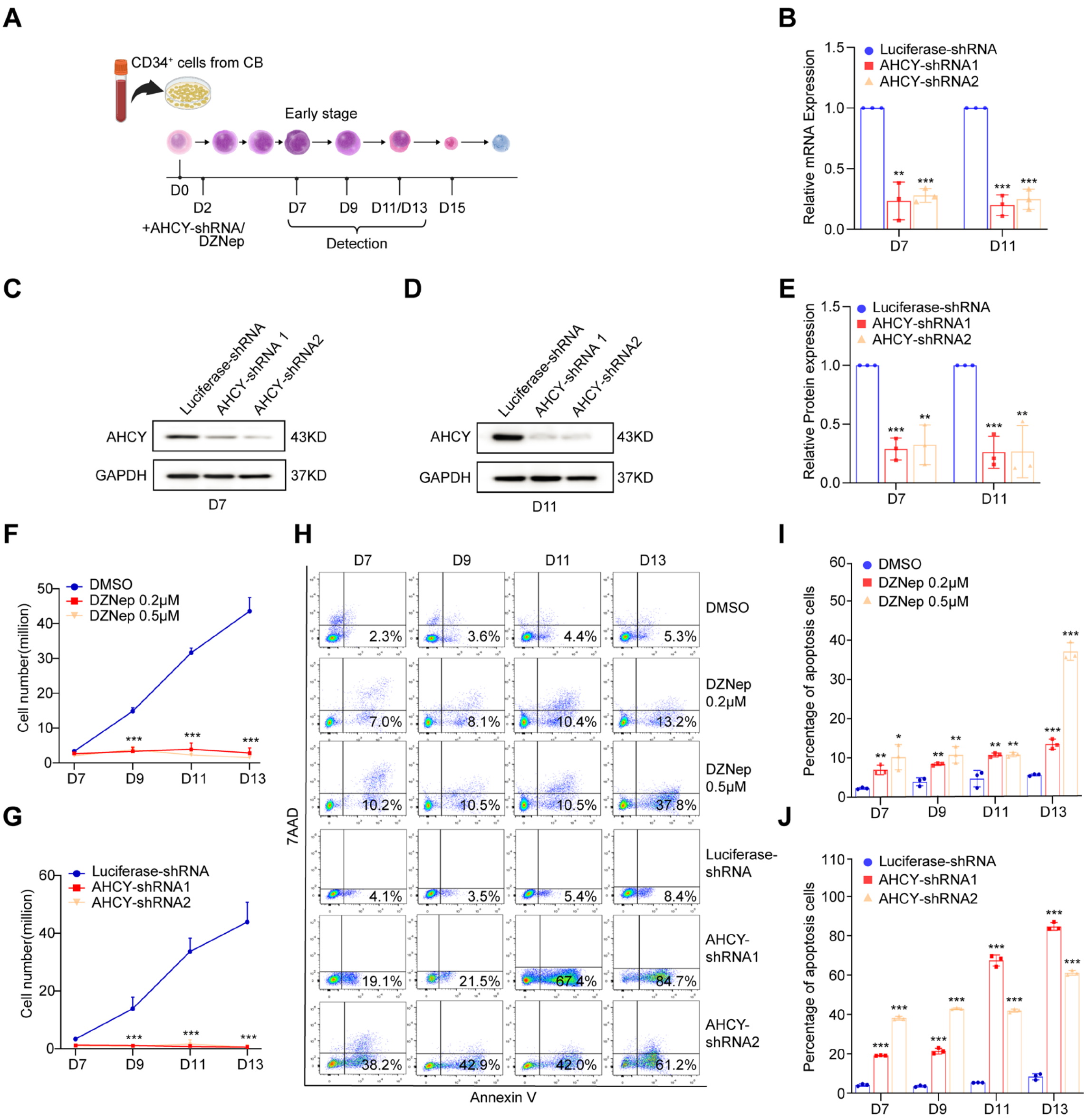

Supplemental Figure 1. AHCY deficiency during early stage of erythropoiesis induces cell apoptosis. A, Schematic diagram of experiment method. The day of getting CD34^{+} cells was recorded as day 0. Lentivirus human CD34^{+}transduction and defunctionized AHCY by treating cells with DZNep at day 2. **B,** Quantitative real-time polymerase chain reaction (qRT-PCR) results showing the knock down efficiency of AHCY in control group and AHCY-shRNA knock down group on days 7 and 11. **C,** Representative western blot results showing the knockdown efficiency of control group and AHCY-shRNA knock down on day 7. **D,** Representative western blot results showing the knockdown efficiency of control group and AHCY-shRNA knock down group on day 11. **E,** Quantitative analysis

the knockdown efficiency of AHCY from 3 independent experiments. **F,** Growth curves of DMSO and DZNep (0.2μM, 0.5μM) treated group determined from fay 7 to day 13. **G,** Growth curves of control and AHCY-shRNA knock down group determined from fay 7 to day 13. **H,** Representative flow cytometry profiles of cells apoptosis determined by double staining with 7-AAD and Annexin V on days 7, 9, 11 and 13 of luciferase control group, AHCY-shRNA knock down group, DMSO group and DZNep (0.2 μM, 0.5 μM) treated group. **I,** Quantitative analysis of ratio of apoptotic cells treated with or without DZNep (0.5 μM, 5 μM) from 3 independent experiments. **J,** Quantitative analysis of ration of apoptotic cells of Luciferase control group and AHCY-shRNA knock down group from 3 independent experiments. Statistical analysis is from 3 independent experiments, and the bar plot represents mean ± standard deviation of triplicate samples. $*P<0.05$, $**P<0.01$, $***P<0.001$.

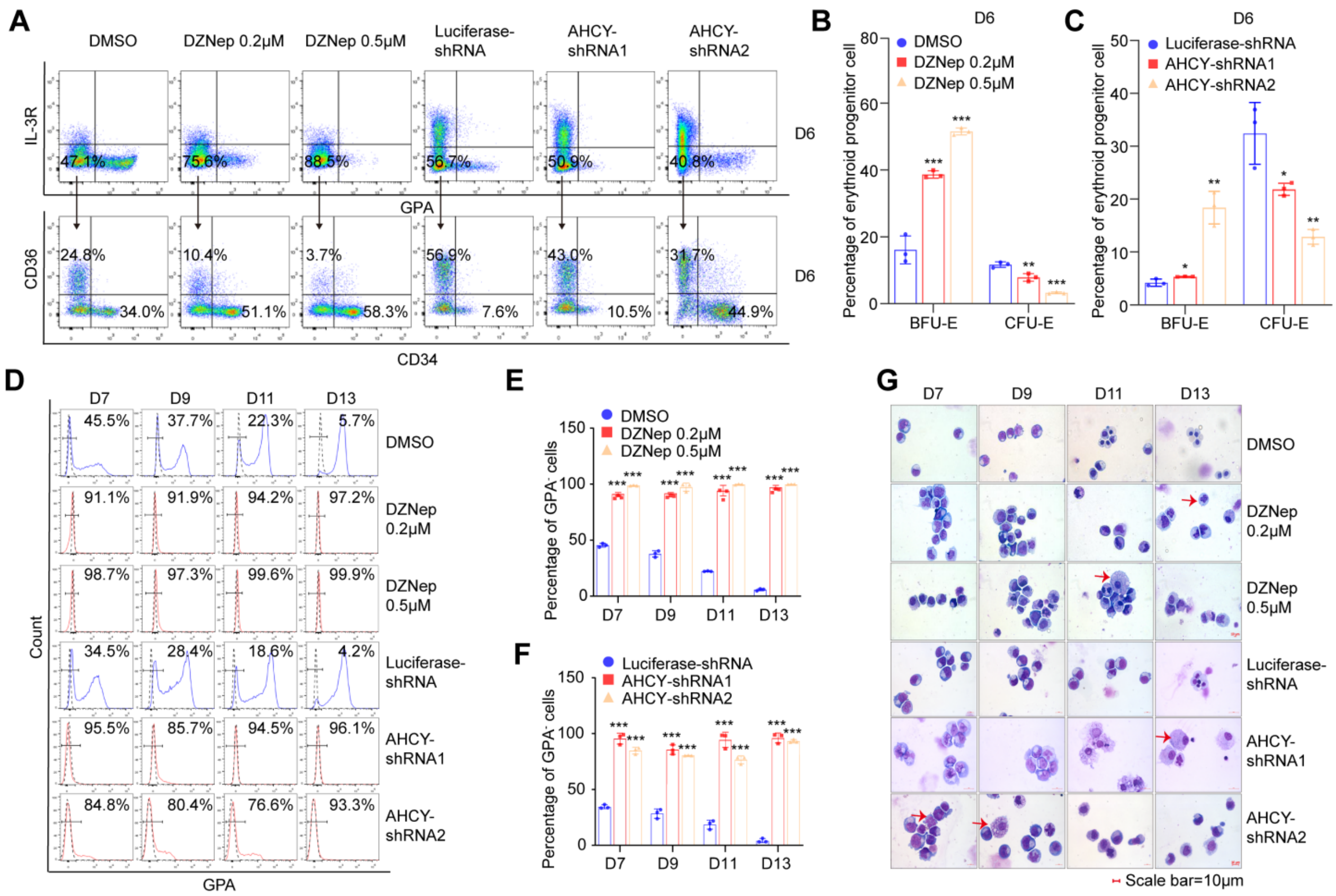

Supplemental Figure 2. AHCY deficiency during early stage of erythropoiesis inhibits erythroid differentiation. A, Flow cytometry analysis of erythroid progenitor cells at day 6. **B**, Statistic analysis of the percentage of BFU-E and CFU-E of DMSO group and DZNep (0.2 µM, 0.5 µM) treated group. **C**, Statistic analysis of the percentage of BFU-E and CFU-E of scramble-shRNA group and AHCY-shRNA group. **D**, Flow cytometry analysis showing the percentage of GPA^- cells on days 7, 9, 11 and 13. **E**, Quantitative analysis of GPA^- cells treated with or without DZNep (0.2 µM, 0.5 µM) from 3 independent experiments. **F**, Quantitative analysis of GPA^- cells of scramble-shRNA and AHCY-shRNA group from 3 independent experiments. **G**, Representative cytospin images of Luciferase-shRNA, AHCY-shRNA, DMSO and DZNep (0.2 µM, 0.5 µM) treated group on day 7, 9, 11 and 13, red arrow points to the cells of the non-erythroid lineage morphology. Scale bar, 10 µm. Statistical analysis is from 3 independent experiments, and the bar plot represents mean ± standard deviation of triplicate samples. *P<0.05, **P<0.01, ***P<0.001.

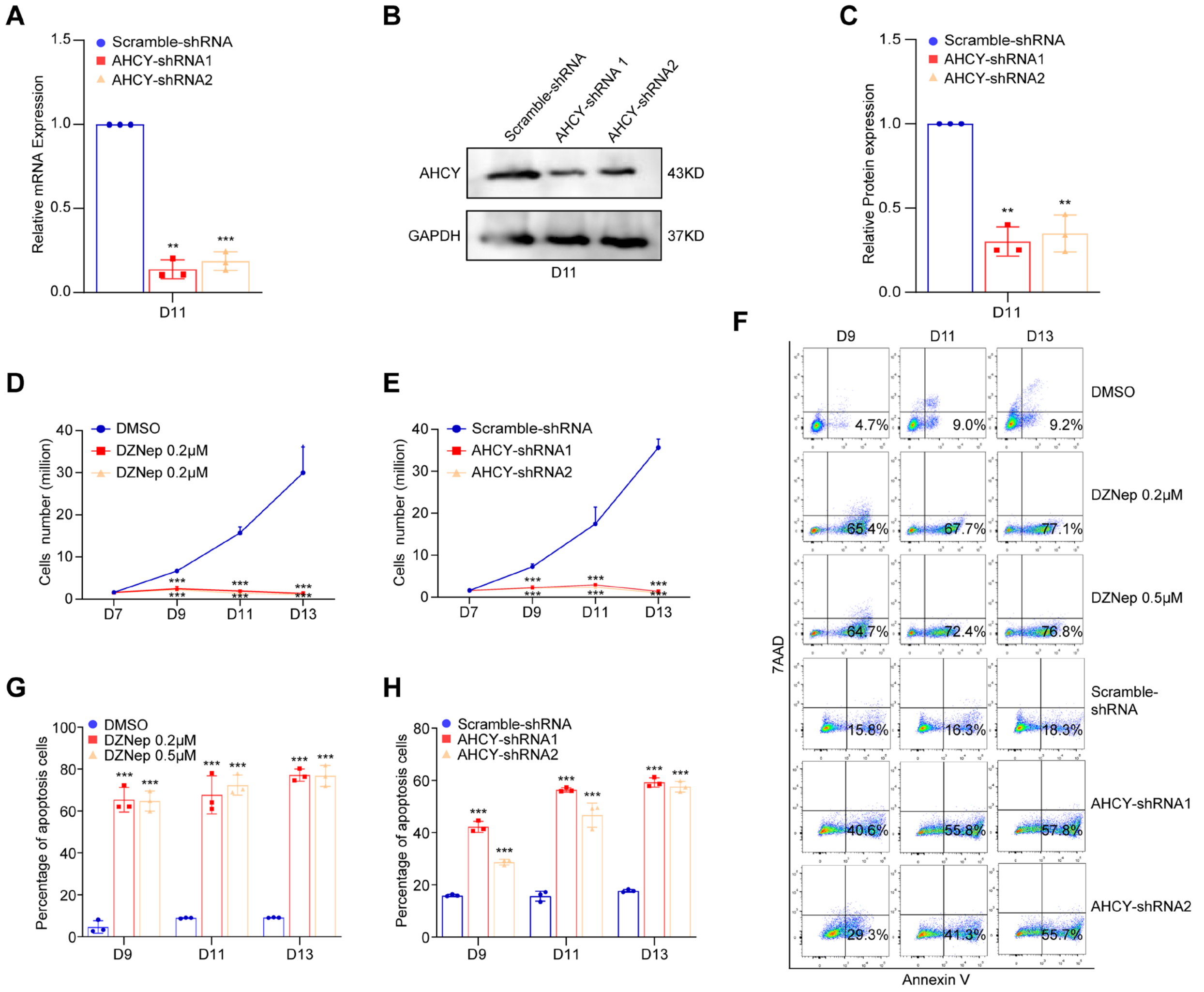

Supplemental Figure 3. AHCY deficiency results in cell apoptosis during terminal stage of erythropoiesis. **A**, qRT-PCR results showing the knock down efficiency of AHCY in Scramble-shRNA and AHCY-shRNA group on days 7 and 11. **B**, Representative western blot results showing the knockdown efficiency of scramble-shRNA and AHCY-shRNA group on day 11. **C**, Quantitative analysis of western blot showing the knockdown efficiency of AHCY from 3 independent experiments. **D**, Growth curves of DMSO and DZNep (0.2μM, 0.5μM) treated group determined from day 7 to day13. **E**, Growth curves of Scramble-shRNA and AHCY-shRNA group determined from day 7 to dan 13. **F**, Representative flow cytometry profiles of cell apoptosis determined by double staining with 7-AAD and Annexin V on days 9, 11 and 13 of Scramble-shRNA, AHCY-shRNA, DMSO and DZNep (0.2 μM, 0.5 μM) treated group. **G**, Quantitative analysis of the ratio of apoptotic cells of control and DZNep (0.2 μM, 0.5 μM) treated group from 3 independent experiments. **H**, Quantitative analysis of the ratio of apoptotic cells of scramble-shRNA and AHCY-shRNA group from 3 independent

experiments. Statistical analysis is from 3 independent experiments, and the bar plot represents mean ± standard deviation of triplicate samples. $^{**}P<0.01$, $^{***}P<0.001$.

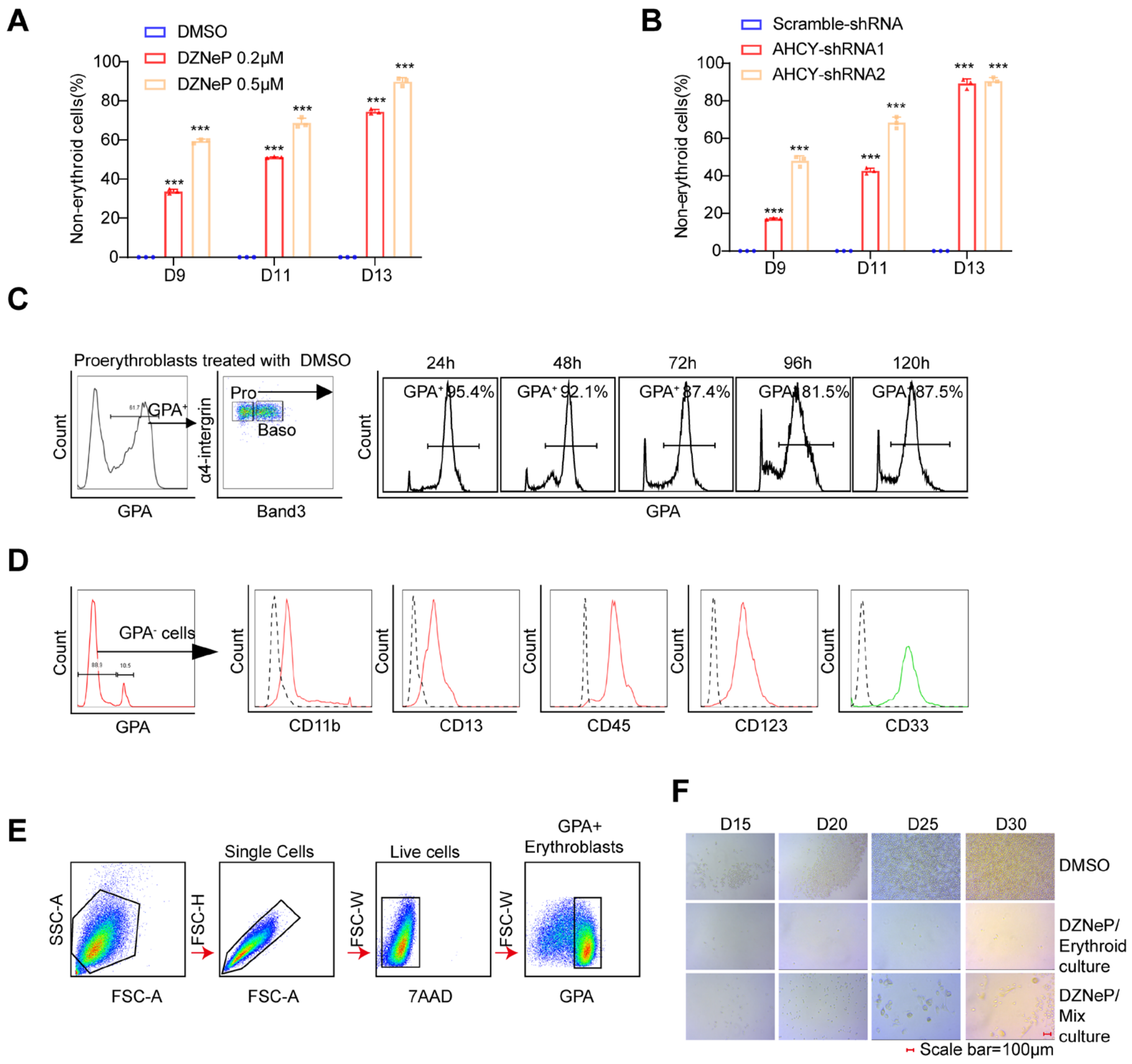

Supplemental Figure 4. AHCY deficiency results in the generation of non-erythroid lineage hematopoietic cells. A, Quantification of non-erythroid lineage cells determined on days 9, 11 and 13 after treatment with DMSO, 0.2 mM DZNeP and 0.5 mM DZNeP at (n=3 independent experiments). **B**, Quantification of non-erythroid lineage cells determined on days 9, 11 and 13 of scramble-shRNA, AHCY-shRNA1, or AHCY-shRNA2 group (n=3 independent experiments). **C**, Proerythroblasts were sorted on day 7 and then treated with DMSO for 72h, 96h and 120h. Flow cytometry analysis showing the percentage of GPA^{+} cells after treatment. **D,** Flow cytometry analysis of surface marker expression on GPA- cells of day 11 after treated with 0.2μM DZNep on day 7. **E,** Representative gating strategy used to sort single GPA^{+} proerythroblasts for downstream treatment. **F,** Microscopic images of cells from three experimental groups acquired on days 15, 20, 25, and 30: DMSO control (cultured in erythroid media); 0.2 μM DZNep-treated (cultured in erythroid media); and 0.2 μM DZNep-treated (cultured in mixed media). Scale bar, 100 μm. The day 30 images for the DMSO (erythroid media),

DZNep (erythroid media), and DZNep (mixed media) groups are reproduced from Figure 1K to illustrate the temporal progression of cell morphology changes. The day 30 panels correspond directly to the images presented in Figure 1K. Data are from 3 independent experiments; bar plots show mean ± SD of triplicate samples. ***P < 0.001.

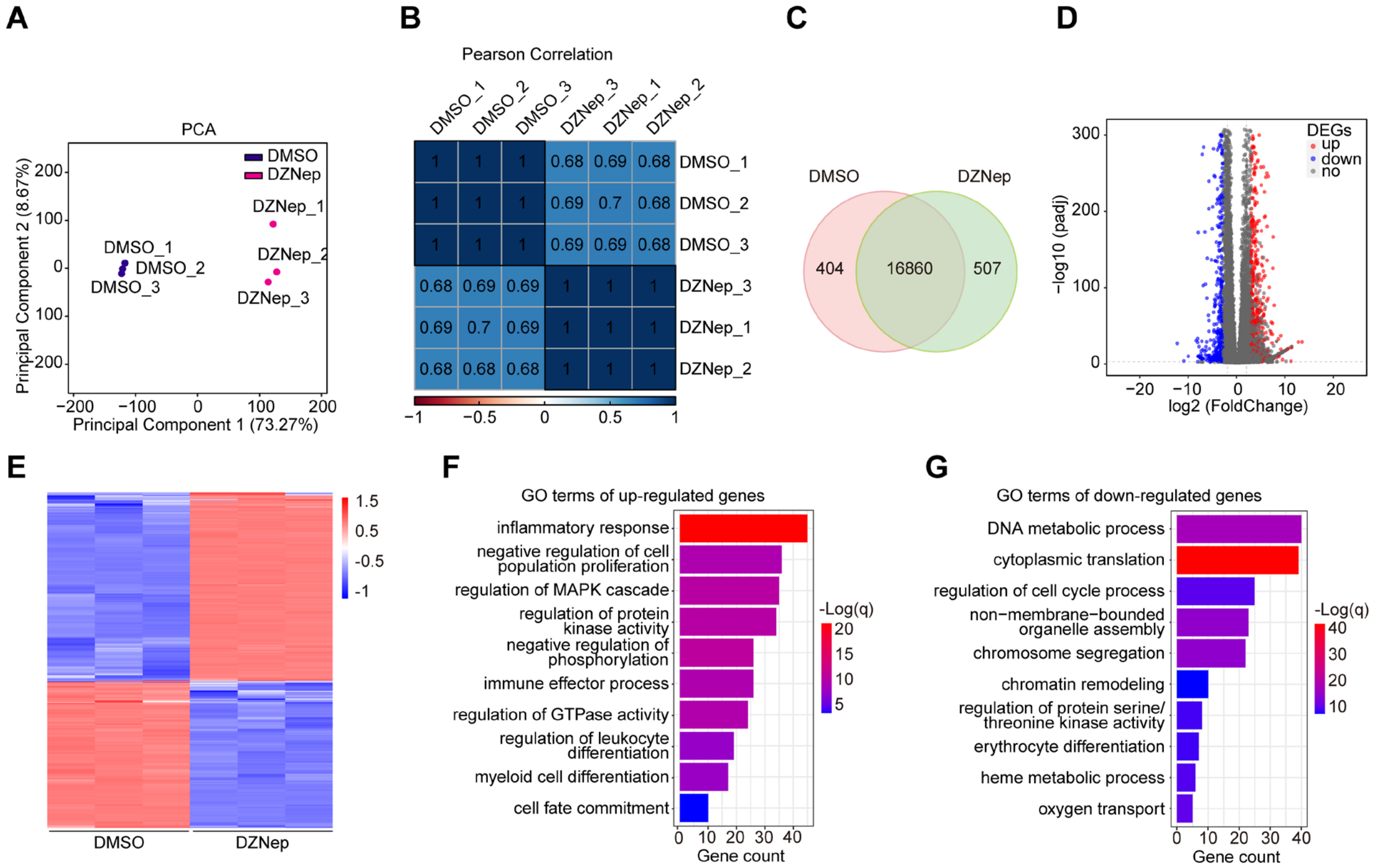

Supplemental Figure 5. RNA-Seq reveals the remodeled expression pattern induced by AHCY deficiency. A, Principal component analysis of samples representing 3 biologic replicates from D11 cells treated with DMSO or 0.2 μM DZNep. **B**, Pearson correlation analysis of RNA-seq. **C**, Venn diagram showing genes with significant difference between DMSO and DZNep treated group. **D**, Volcano map showing genes with significant difference between DMSO and DZNep treated group. **E**, Heat map showing expression values of differentially expressed genes (DEGs) between DMSO and DZNep treated group. **F**, The top upregulated pathways revealed by gene ontology gene ontology (GO) analysis of the differentially expressed genes between DMSO and DZNep treated group. **G**, The top downregulated pathways revealed by gene ontology gene ontology (GO) analysis of the differentially expressed genes between DMSO and DZNep treated group.

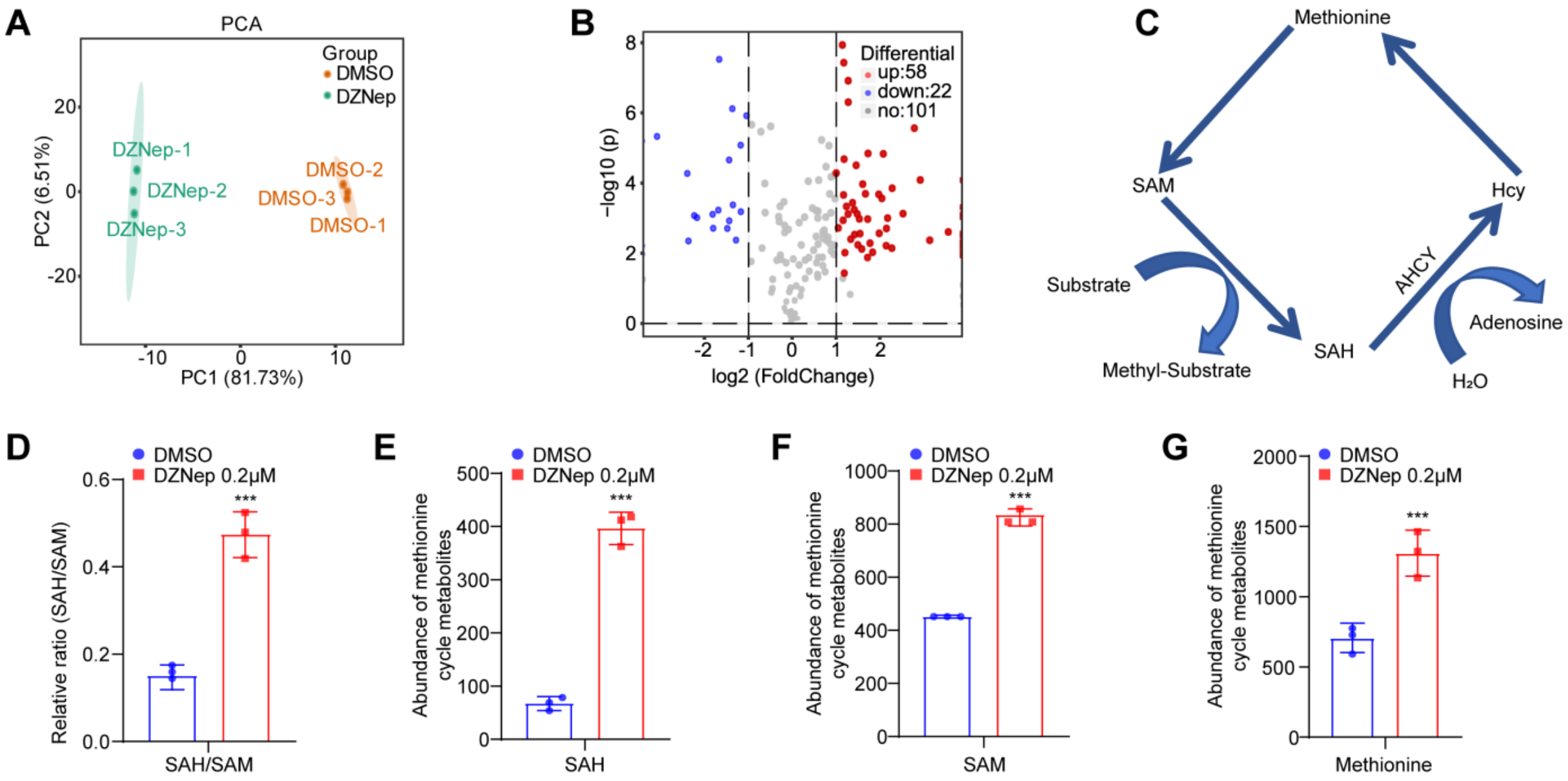

Supplemental Figure 6. AHCY dysfunction disrupts methionine metabolism in erythroblasts. **A**, Principal component analysis of samples representing 3 biologic replicates from D11 cells treated with 0.2 µM DZNep. **B**, Volcano map showing the relative abundances of each metabolite in each sample. **C**, Schematic overview of methionine metabolism. **D**, Relative SAH to SAM ratio analyzed by LC-MS in Day 11 erythroblasts. Ratio was calculated from raw peak areas measured by LC-MS/MS in MRM mode. **E-G**, Intracellular SAH, SAM and methionine concentrations detected by LC-MS.

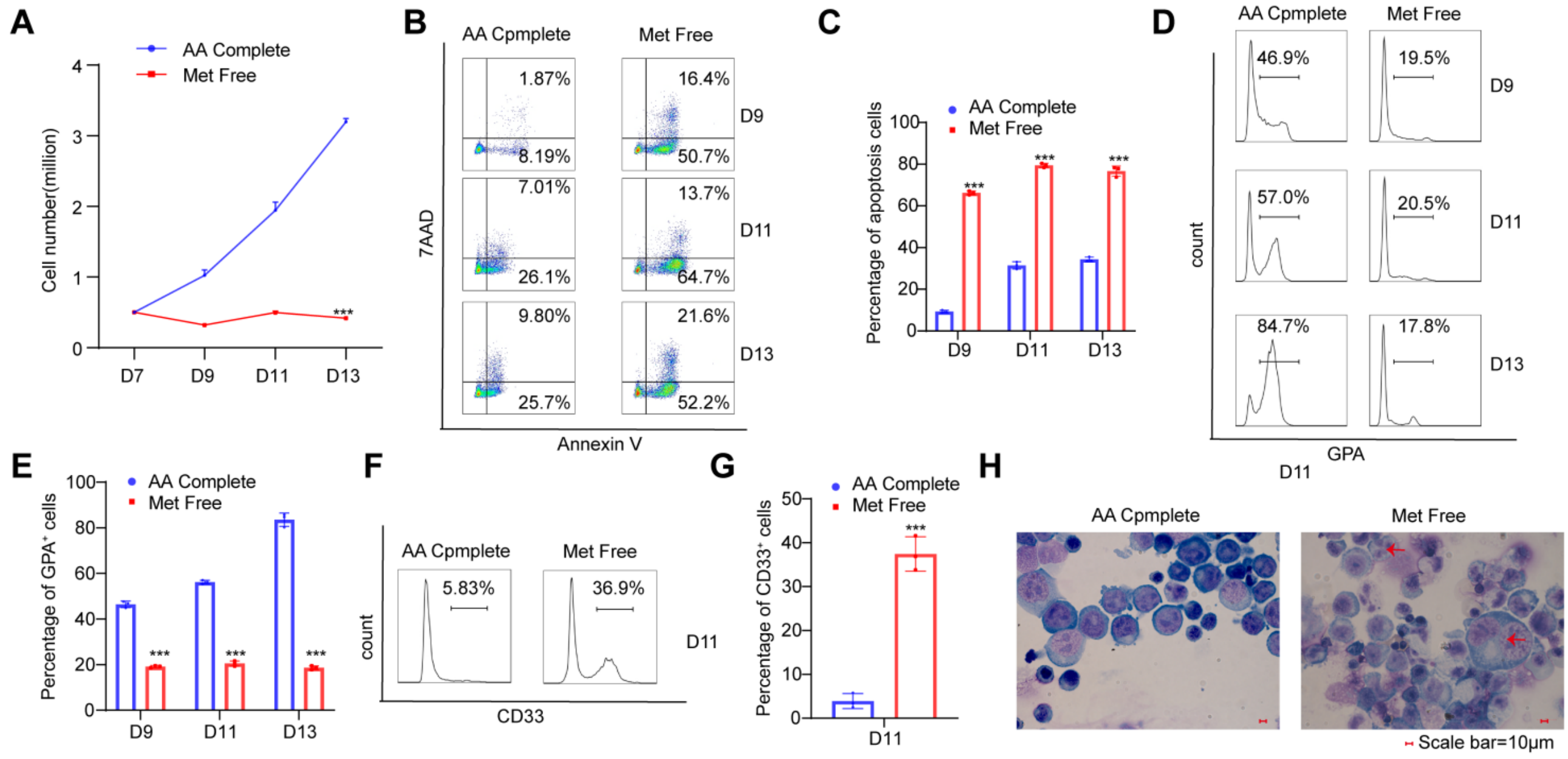

Supplemental Figure 7. Methionine deprivation induces cell apoptosis and cell fate reprogramming in terminal erythroblasts. A, Growth curves of cells cultured in amino acid complete medium (AA Complete) or methionine-free medium (Met Free) from day 7 to day 13. **B**, Representative flow cytometry plots of apoptosis determined by 7-AAD and Annexin V double staining on days 9, 11, and 13 for AA Complete and Met Free groups. **C**, Quantitative analysis of apoptotic cell percentages from 3 independent experiments. **D**, Flow cytometry analysis showing the percentage of GPA^+ cells on days 9, 11 and 13. **E**, Quantitative analysis of GPA^+ cell percentages from 3 independent experiments. **F**, Flow cytometry analysis showing the percentage of $CD33^+$ cells on day 11. **G**, Quantitative analysis of $CD33^+$ cell percentages from 3 independent experiments. **H**, Representative cytospin images of AA Complete and Met Free group on day 11. Red arrows indicate cells with non-erythroid morphology. Scale bar, 10 μm. Data are presented as mean ± SD from three independent experiments. $^{**}p < 0.01$, $^{***}p < 0.001$

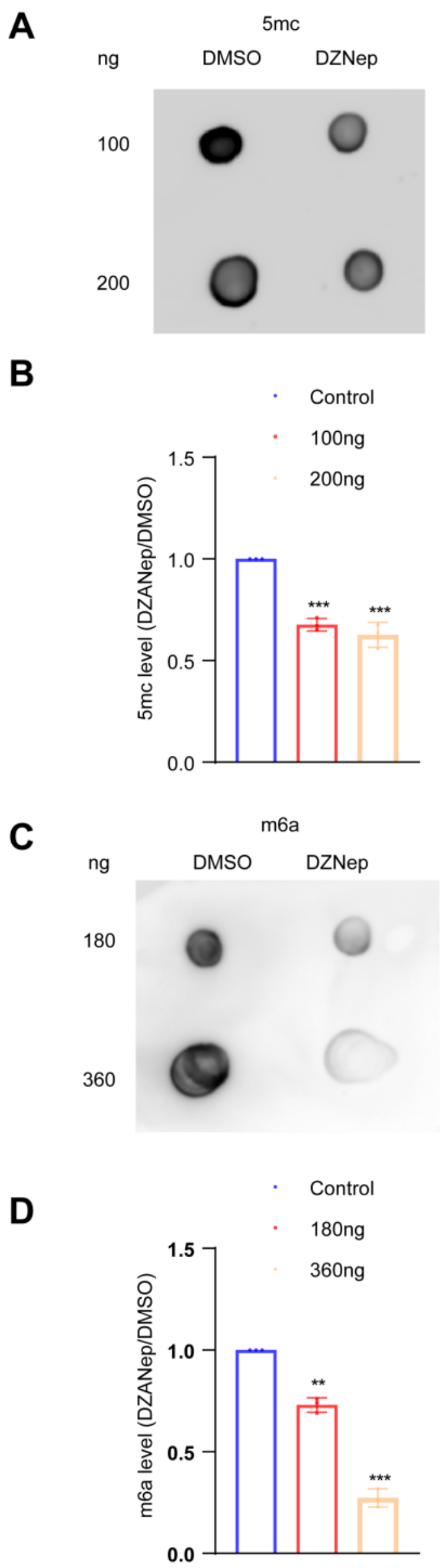

Supplemental Figure 8. DNA and RNA methylation are decreased in AHCY deficient proerythroblasts. A, Representative dot blot results showing 5mC levels in genomic DNA from DMSO control and DZNep-treated cells (100 ng and 200 ng). **B**, Quantification of 5mC dot blot signals from 3 independent experiments. **C**, Representative dot blot results showing m^6A levels in total RNA from DMSO control and DZNep-treated cells (180 ng and 360 ng). **D**, Quantification of m^6A dot blot signals from 3 independent experiments. Data are presented as mean ± SD of triplicate samples. $^{**}P<0.01$, $^{***}P<0.001$.

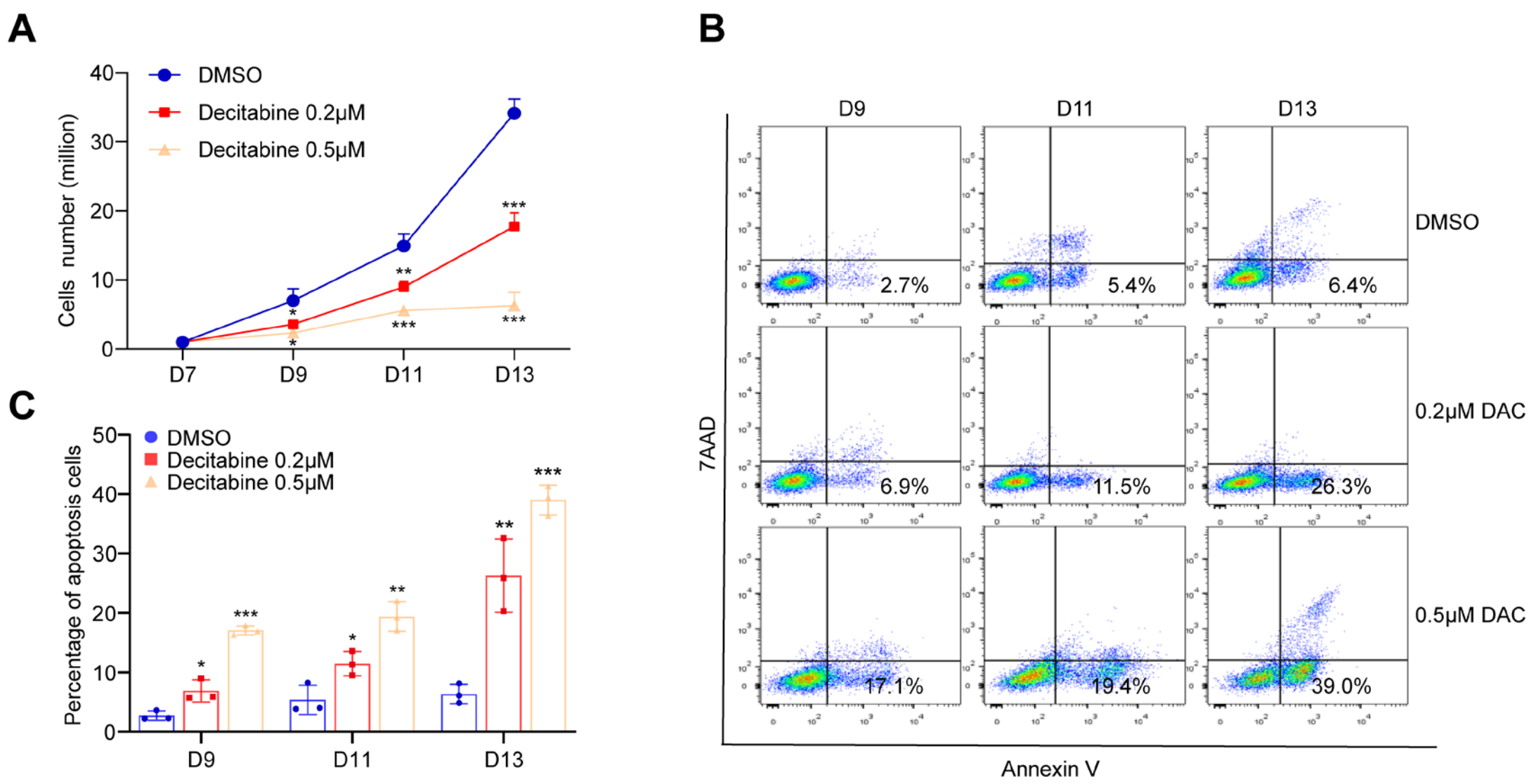

Supplemental Figure 9. Inhibition of DNA methylation does not affect terminal erythroid differentiation. A, Growth curves of DMSO group and Decitabine (0.2 µM, 0.5 µM) treated group. **B**, Representative flow cytometry profiles of the ration of apoptosis cells determined by double staining with 7-AAD and Annexin V on days 9, 11 and 13 of control group and Decitabine (0.2 µM, 0.5 µM) treated group. **C**, Quantitative analysis of the ration of apoptotic cells from 3 independent experiments. Statistical analysis is from 3 independent experiments, and the bar plot represents mean ± standard deviation of triplicate samples. *P<0.05, **P<0.01, ***P<0.001.

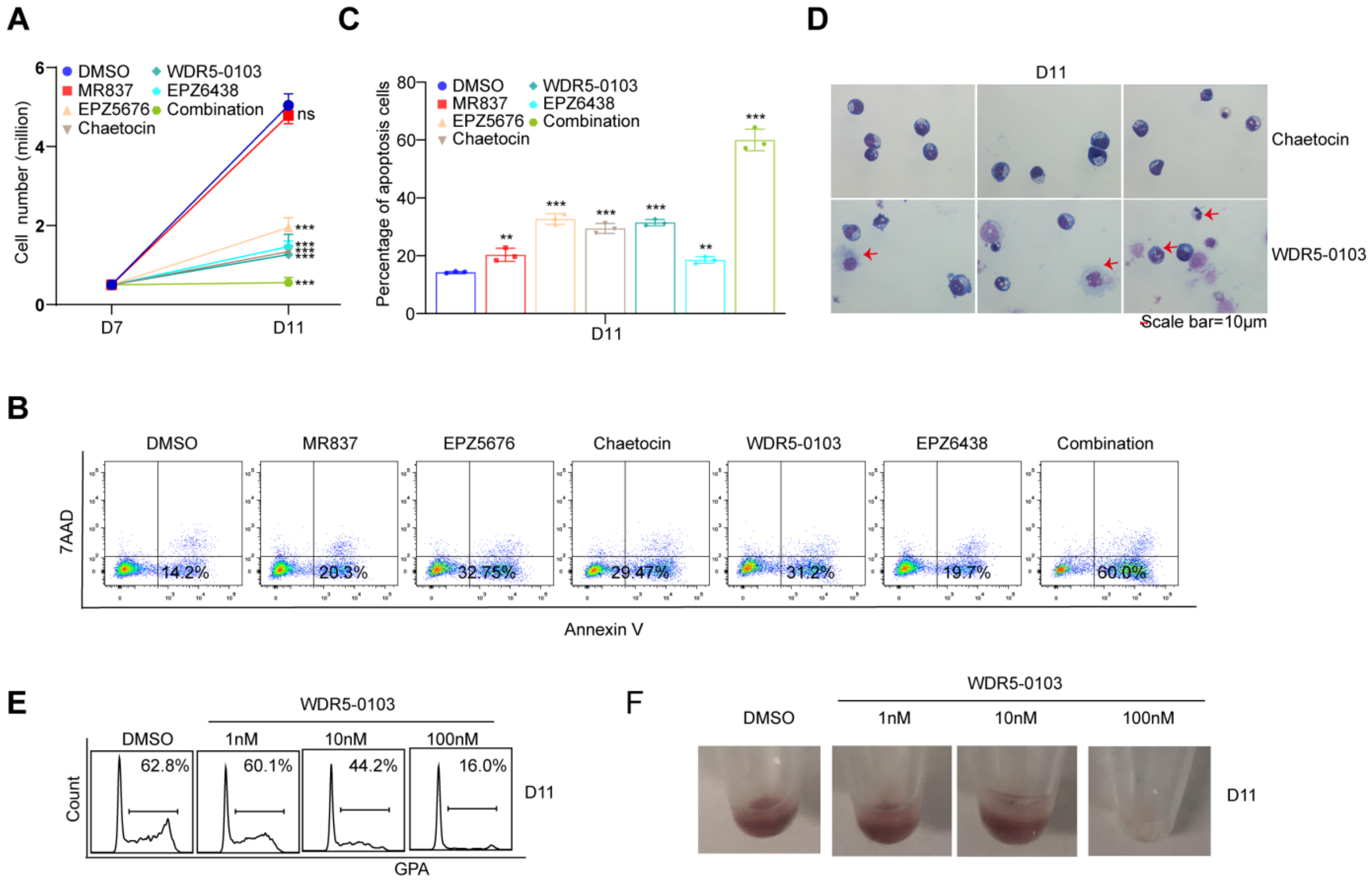

Supplemental Figure 10. Inhibition of histone methylations during terminal stage impairs cell growth and apoptosis. A, Growth curves of DMSO group and various types of histone methylation inhibitors treated group. **B**, Representative flow cytometry profiles showing the cell apoptosis determined by double staining with 7-AAD and Annexin V on day 11 of after treatment. **C**, Quantitative analysis showing the percentage of apoptotic cells from 3 independent experiments. **D**, Representative cytospin images of cells after treatment with Chaetocin and WDR5-0103, red arrow pointed to the morphological identified non-erythroid lineage cells. Scale bar, 10 μm. **E**, Flow cytometry analysis showing the percentage of GPA^+ cells after treatment with WDR5-0103. **F**, Representative cell pellet of WDR5-0103 treated group. Statistical analysis is from 3 independent experiments, and the bar plot represents mean ± standard deviation of triplicate samples. $^{**}P<0.01$, $^{***}P<0.001$.

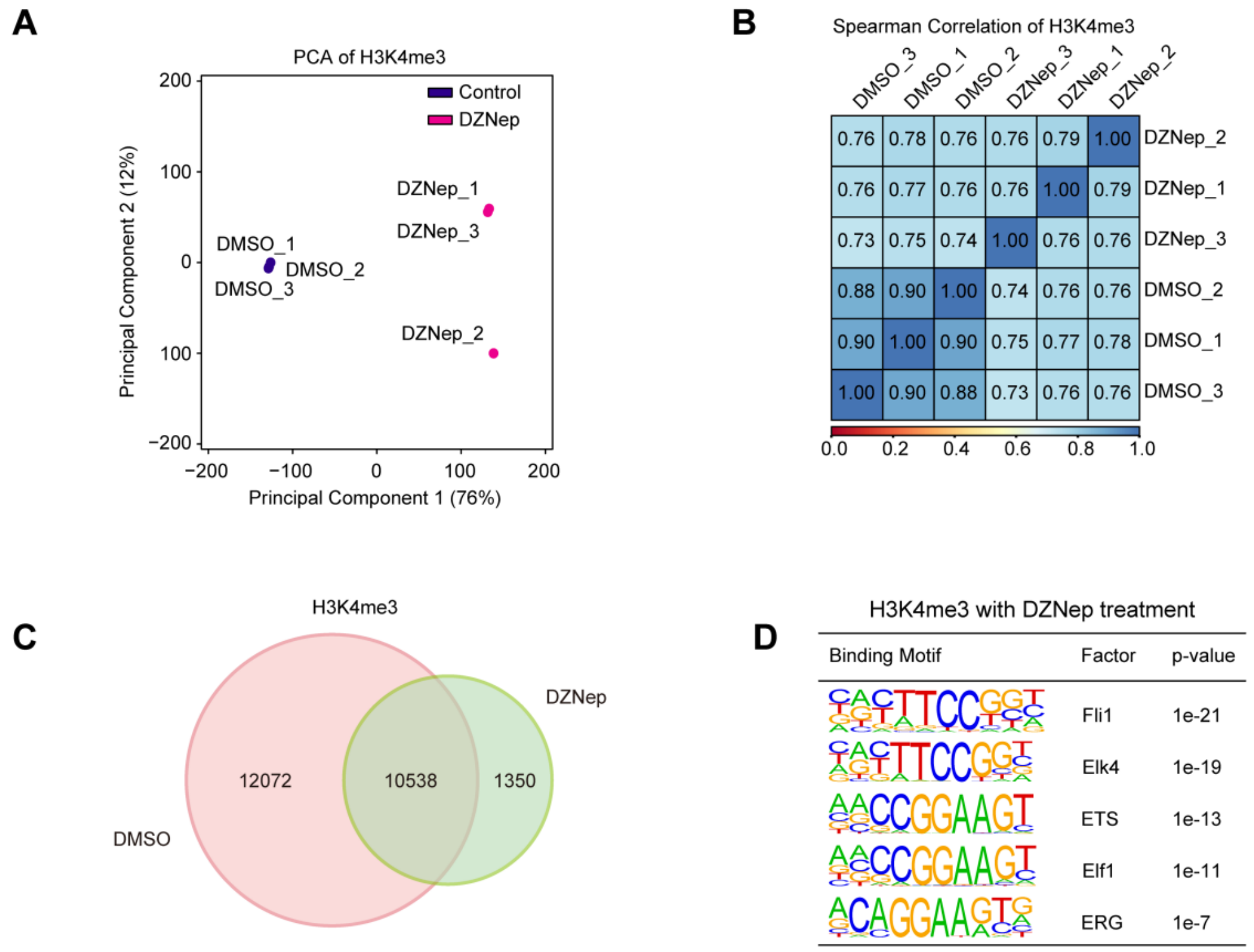

Supplemental Figure 11. CUT&Tag sequencing analysis of H3K4me3 distribution. A, Principal component analysis of samples representing 3 biologic replicates from D11 cells treated with 0.2 μM DZNep. **B,** Spearman correlation analysis of CUT&Tag. **C,** Venn diagram showing differences in H3K4me3 CUT&Tag peak between the DMSO and DZNep groups. **D,** Top TF motifs enriched in H3K4me3 CUT&Tag peaks linked to promoters after DZNep treatment.

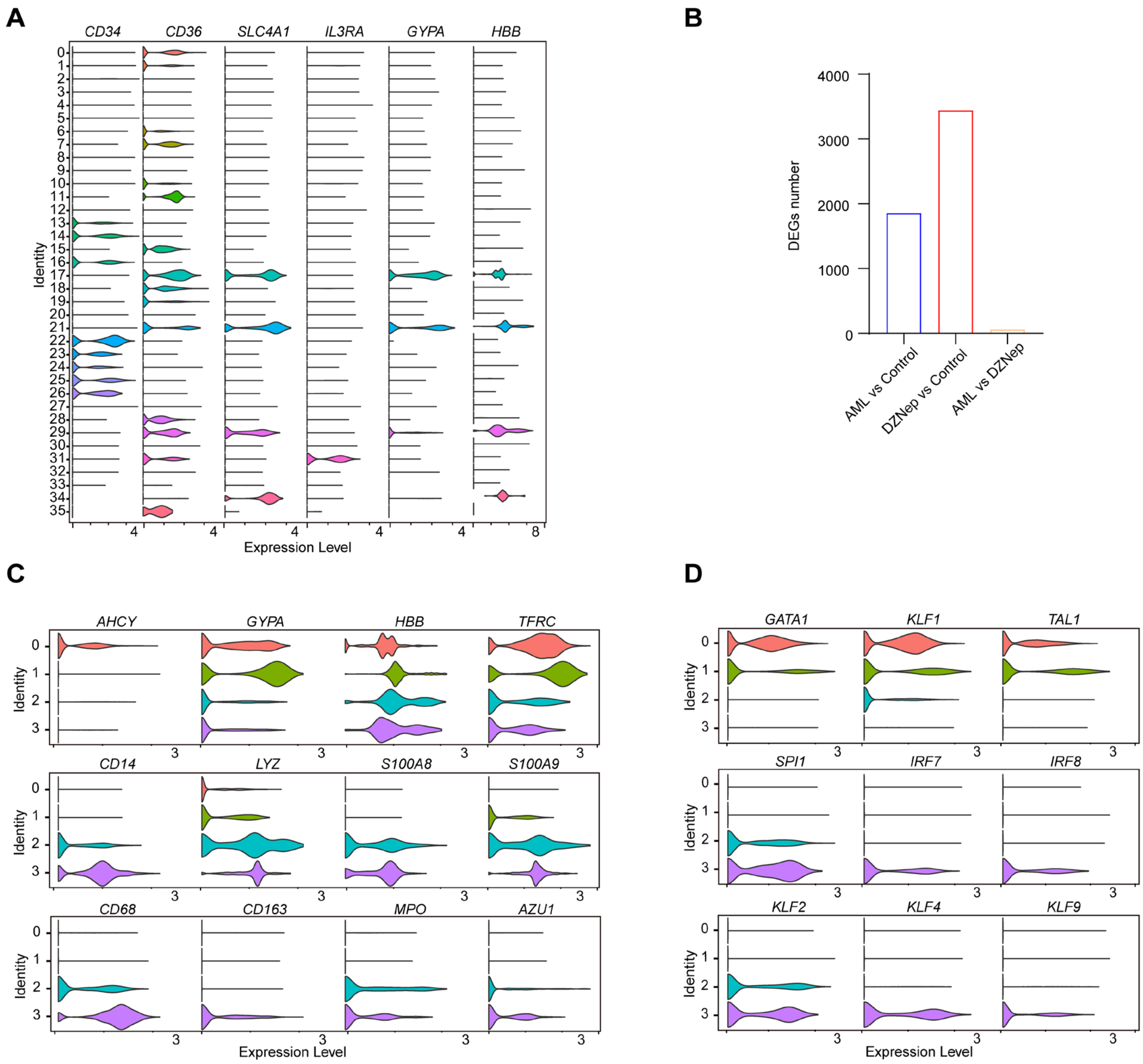

Supplemental Figure 12. Erythroblasts from AML patients and those treated with DZNep share similar transcriptome characteristics. A, Violin plots depicting the mRNA expression in each subcluster. **B,** Statistics on the number of DEGs of AML, healthy controls and DZNep-treated erythroblasts. **C,** Violin plots depicting the expression of several genes that are usually co-expressed in erythroblasts of three groups (*TFRC*, *GYPA*, *HBB*), high expressed in erythroblasts of healthy individuals (*AHCY*), or low expressed in erythroblasts of healthy individuals (all other genes). **D,** Violin plots depicting the expression of erythroid-specific transcription factors that are high expressed in cluster 0 of erythroblasts (*GATA1*, *KLF1*, *TAL1*), or other lineage-specific transcription factors are high expressed (all other genes).

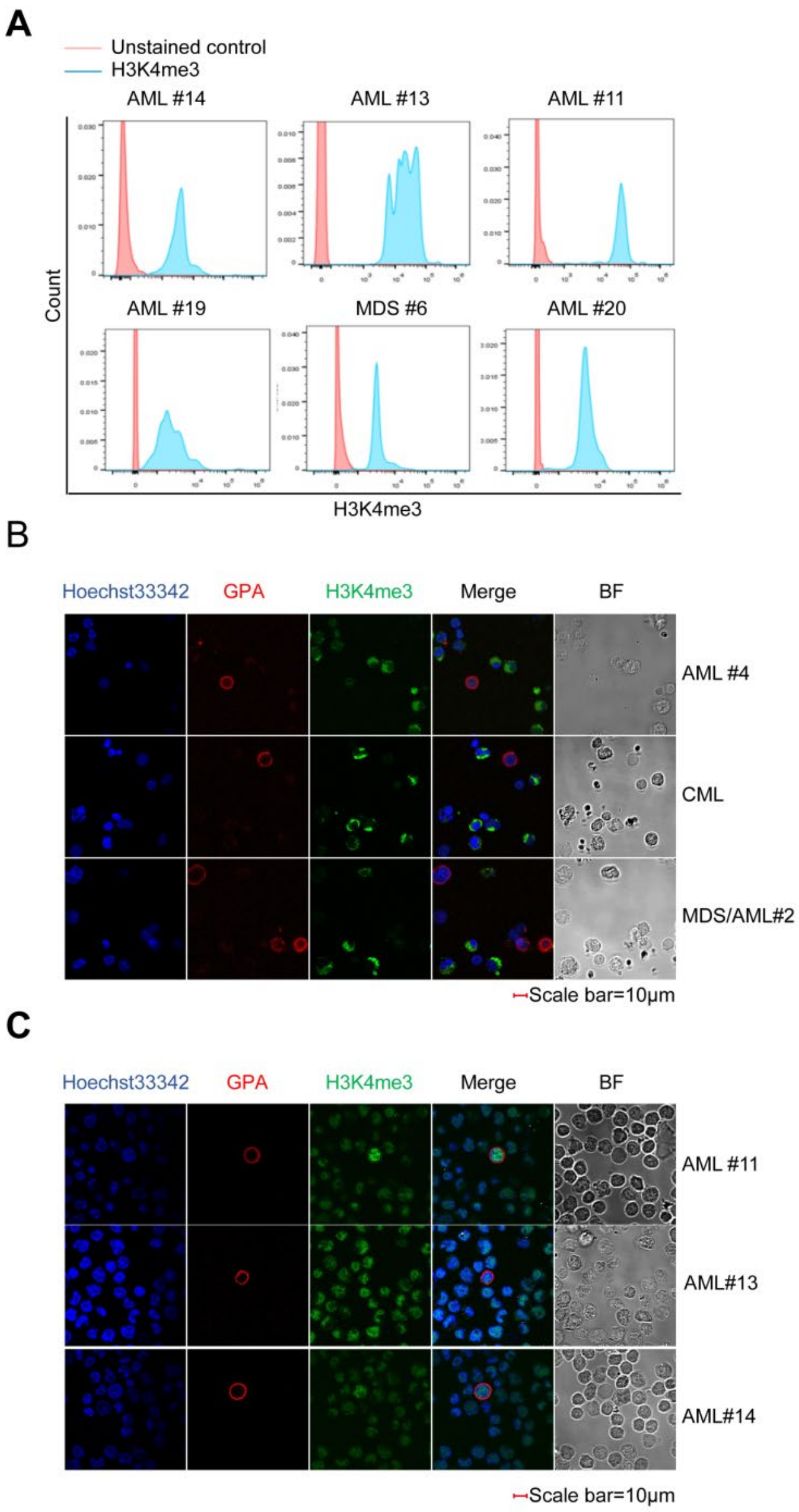

Supplemental Figure 13. Reduced existence of H3K4me3 in erythroblast of bone marrow from AML patients. A, Flow cytometry analysis showing the reduced level of H3K4me3 in erythroblasts from 9 out of 39 myeloid leukemia patients. **B**, Immunofluorescence images showing the reduced level of H3K4me3 (green) in erythroblasts from the other 30 myeloid leukemia patients. Hoechst 33342 (blue) was used to stain the nucleus. GPA (red) was stained to display the erythroblast membrane. Scale bar, 10 μm. **C**, Immunofluorescence images showing the normal level of existence of H3K4me3 in erythroblasts (green) from leukemia patients. Hoechst 33342 (blue) was used to stain the nucleus. GPA (red) was stained to display the erythroblast membrane. Scale bar, 10 μm.

Supplemental Table 1. Antibodies and reagents

antibody	clone	conjugate	Catlog#	company
Mouse monoclonal anti-human band 3	no	FITC	N/A	Generated in our laboratory
anti-human CD235a/GPA	GA-R2	APC	551336	BD Biosciences
anti-human α4 integrin	MZ18-24A9	APC	130-124-008	MACS
anti-human CD34	581	PE	555822	BD Biosciences
anti-human CD36	CB38	FITC	555454	BD Pharmingen
anti-human CD123 (IL-3R)	6H6	PE-Cy7	25-1239-42	Invitrogen
anti-human CD45	2D1	APC/Cyanine 7	368515	biolegend
Annexin V	no	APC	88-8103-74	eBioscience
Hoechst33342	no	no	B8040	Solarbio
7AAD	no	no	00-6993-50	eBioscience
Mouse anti-human GAPDH antibodies	1D4	no	sc-59540	Santa Cruz
anti-human AHCY antibodies	34h5	no	RN126PW	MBL
anti-Histone H3	D1H2	no	# 60932	Cell Signaling Technology
anti-H3K4me3	C42D8	PE	# 9751	Cell Signaling Technology
anti-H3K9me3	D4W1U	APC	# 13969	Cell Signaling Technology
anti-H3K27me3	C36B11	no	# 9733	Cell Signaling Technology
anti-H3K36me2	C75H12	no	# 2901	Cell Signaling Technology
anti-H3K79me2	D15E8	no	# 5427	Cell Signaling Technology
Rabbit (DA1E) mAb IgG XP® Isotype Control antibodies	DA1E	PE-Cy7	# 3900	Cell Signaling Technology
Ter119 antibody	TER-119	biotin	13-5921-85	ebioscience
mouse CD45 antibody	30-F11	biotin	13-0451-82	ebioscience

Supplemental Table 2. Sequences of the primers and shRNA

Name	Sequence/Catalog Number
Forward primer of AHCY for qRT-PCR	ATTCCGGTGTATGCCTGGAAG
Reverse primer of AHCY for qRT-PCR	GAGATGCCTCGGATGCCTG
Forward primer of GAPDH for qRT-PCR	GGAGTCCACTGGCGTCTTCA
Reverse primer of GAPDH for qRT-PCR	GTCATGAGTCCTTCCACGATACC
Forward primer of GATA1 for qRT-PCR	ATCACACTGAGCTTGCCACA
Reverse primer of GATA1 for qRT-PCR	CAGGCCAGGGAACTCCA
Forward primer of GYPA for qRT-PCR	GGGTGATGGCTGGTGTTATTGGAACGATC
Reverse primer of GYPA for qRT-PCR	GGCACGTCTGTGTCAGGTGAGGGGAG
Forward primer of KLF1 for qRT-PCR	CACACAGGATGACTTCCTCAA
Reverse primer of KLF1 for qRT-PCR	GCTGGTCCTCAGACTTCACG
Forward primer of GATA2 for qRT-PCR	GCAACCCCTACTATGCCAACC
Reverse primer of GATA2 for qRT-PCR	CAGTGGCGTCTTGGAGAAG
Forward primer of CEBPA for qRT-PCR	TGAGTGAGGCTCTCATTCTT
Reverse primer of CEBPA for qRT-PCR	ACATACACCCTTGGACAACTA
Forward primer of KIT for qRT-PCR	GGGCCACCGTTTGGAAAG
Reverse primer of KIT for qRT-PCR	TTACATTCAACCGTGCCATTG

AHCY-shRNA1	CCGGATGCCATTGTGTGTAACATTGCTCG AGCAATGTTACACACAATGGCATTTTTG
AHCY-shRNA2	CCGGGTCAGGAGGGCAACATCTTTGCTCG AGCAAAGATGTTGCCCTCCTGACTTTTTG

Supplemental Table 3. Small molecule epigenetic modification inhibitors

Chemicals	Catlog#	company	Target	Histone Modification	Working concentration	MW
3-Deazaneplanocin A (DZNep)	102052-95-9	MCE	AHCY	N/A	0.2μM and 0.5μM	262.26
Decitabine (5-Aza-2'-deoxycytidine)	2353-33-5	MCE	DNA methyltransferase	N/A	0.2μM and 0.5μM	228.21
EPZ5676	S7062	Selleckchem	DOT1L	H3K79Me2	10μM	562.71
Chaetocin	S8068	Selleckchem	SU(VAR)3-9	H3K9Me3	0.05μM	696.84
EPZ6438	S7128	Selleckchem	EZH2	H3K27Me3	0.2μM	572.74
MR837	1210906-48-1	MCE	NSD2-PWWP1	H3K36Me2	3.4μM	282.36
WDR5-0103	890190-22-4	MCE	WDR5	H3K4Me3	100nM	383.44

Supplemental Table 4. Patient samples (at diagnosis) used in this study

SAMPLE	% of GPA$^+$ cells in PBMC	H3K4me3 heterogeneity information	Clone formation information
Healthy control#1	8.39%	no	no
MDS#1		no	no
AML#1	0.97%	no	no
AML#2	8.22%	no	no
AML#3	0.33%	no	no
AML#4	0.71%	yes	yes
AML#5	1.00%	no	yes
AML#6	2.58%	yes	yes
CML	1.73%	yes	yes
MDS/AML#1	4.61%	yes	no
AML#7	1.03%	yes	yes
AML#8	3.14%	yes	no
AML#9	11.70%	yes	no
AML#10	0.39%	no	yes
Healthy control#2	1.14%	no	no
AML#11	3.62%	yes	no
AML#12	9.96%	no	no
MDS/AML#2	2.33%	yes	yes
AML#13	0.99%	no	no
AML#14	1.77%	yes	no
AML#15	3.22%	yes	yes
AML#16	2.39%	no	no
MDS#2	1.68%	no	no
AML#17	2.33%	yes	yes
AML#18	2.43%	yes	yes
MDS#3	7.82%	no	no
MDS#4	4.22%	no	no
AML#19	0.53%	no	no
AML#20	0.28%	no	no
AML#21	0.71%	yes	yes
MDS#5	2.17%	yes	no
MDS#6	0.86%	no	no

Drug treatment

The drugs used for treating cells were as following: 3-Deazaneplanocin A (DZNep) was purchased from MCE and adding in cell culture medium at a final concentration of 0.2μM and 0.5μM. Decitabine (5-Aza-2 -deoxycytidine) was purchased from MCE, which was dissolved in DMSO and using at a final concentration of 0.2μM and 0.5μM. Tazemetostat (EPZ-6438, S7128) was purchased from Selleck and adding in cell culture at a final concentration of 0.2 μM. Pinometostat (EPZ5676, S7062) was purchased from Selleck and adding in cell culture at a final concentration of 10 μM. Chaetocin (S8068) was purchased from Selleck and adding in cell culture at a final concentration of 0.05 μM. MR837 was purchased from MCE and adding in cell culture at a final concentration of 3.4 μM. WDR5-0103 was purchased from MCE and adding in cell culture at a final concentration of 100 nM (Supplementary Table 3).

CUT&Tag

Cells were harvested, counted and centrifuged for 3 min at 600 ×*g* at room temperature. 1×10^5 cells were washed twice in 500 μL wash buffer by gentle pipetting. Concanavalin A coated magnetic beads were prepared as the kit manual described, 10 μL of activated beads were added per sample and incubated at RT for 10 min. According to the manufacture instructions, cells were sequentially incubated with ConA Beads, primary antibody (anti-H3K4me3), secondary antibody (Goat anti-Rabbit IgG) and Hyperactive PG-TN5/PA-TN5 Transposon and then fragmented. The fragmented DNA was extracted from the samples and amplified by PCR. CUT&Tag libraries were constructed using HyperactiveTM In-Situ CUT&Tag Library Prep Kit (TD901, Vazyme, China) and sequenced on an Illumina NovaSeq platform, and 150-bp paired-end reads were generated. Trim Galore version 0.6.6 was used to remove adapter and low-quality reads. Align paired-end reads using Bowtie2 version 2.5.1

with options: “--end-to-end --very-sensitive”. Peak calling uses MACS2 version 2.1.1 with threshold: $q<0.01$, and the differential binding sites of H3K4me3 were calculated using the DiffBind version 3.8.4 with the parameter log2foldchange >=1 and FDR <0.05 (https://www.cruk.cam.ac.uk/core-facilities/bioinformaticscore/software/diffbind.html). Scatterplots, correlation plots, and heatmaps are displayed using deepTools version 2.5.7. Annotation of peaks is performed using an R package ChIPseeker version 1.34.1(https://www.cruk.cam.ac.uk/core-facilities/bioinformatics-core/software/ChIPseeker.html). The CUT&Tag peak/region <1000bp from the nearest TSS was defined as the promoter. Hypergeometric Optimization of Motif Enrichment (HOMER) version 4.11 (http://homer.ucsd.edu/homer/) was used to search for the binding motif.

RNA-seq and bioinformatics analysis

Experiment procedure before sequencing: 2×10^6 cells were collected and washed twice with PBS. RNA was extracted and used as cDNA input. cDNA library was prepared by Illumina Truseq DNA library kit, and sequenced on Illumina NovaSeq 6000. The original data were filtered and compared with the reference genome, which can be downloaded from the Ensemble database (http://www.ensembl.org/index.html,human genome build GRCh38.87). Using HISAT2 version 2.2.1 (http://daehwankimlab.github.io/hisat2/) to read the counts for each gene in each sample and gene expression was assessed by StringTie (https://ccb.jhu.edu/software/stringtie/). TPM (Transcripts Per Kilobase of exon model per Million mapped reads) was calculated to evaluate the expression level, and DESeq2 version 1.38.3 (https://bioconductor.org/packages/release/bioc/html/DESeq2.html) was packaged to identify differentially expressed genes from the data. Gene ontology (GO) enrichment analysis and visualization of DEGs was performed using the Metascape (www.metascape.org/) online tool with $q < 0.05$.

Single-cell RNA sequencing (scRNA-seq)

Adherent and suspended cells were collected after treatment with 0.5 μM DZNep and used for single-cell suspension preparation and quality inspection. According to the library construction process officially recommended by 10× Genomics, library construction and on-board sequencing were performed. After obtaining the expression matrix through the Cellranger version 6.0.1 (https://www.10xgenomics.com/), low-quality cells were filtered, and PCA was applied for dimensionality reduction of the highly variable gene expression matrix. Using a graph-based clustering algorithm, the clustering results were also visualized with UMAP in two dimensions. Cell annotation was followed using the R package SingleR version 2.5.0 (https://bioconductor.org/packages/devel /bioc/html/SingleR.html). Cells of interest were selected for subset analysis. Next, single-cell trajectory analysis was performed using the R package Monocle3 version 1.3.1 (https://bioconductor.org/packages/ devel/bioc/html/monocle.html).

Regulatory network analysis

pySCENIC version 0.10.4 (https://aertslab.org/#scenic) was used to construct the cell typespecific GRNs in the integrated dataset of erythroid cell treated with DZNep. After inputting the single-cell expression profiles, the co-expression modules were analyzed using the RcisTarget package, and cis-regulatory motifs were identified based on human databases (hg38_refseqr80_10kb_up_and_down_ tss.mc9nr.feather). Next, the enrichment of a regulon was measured according to the area under the recovery curve, as determined using the AUCell package. The regulatory factors for each cell cluster are presented in R package pheatmap version 1.0.12 (https://bioconductor.org/packages/release/bioc /html/heatmaps.html).

Re-analysis of scRNA-seq data

The cell-by-gene count matrix data for each sample generated using the 10× Genomics platform, was downloaded from Gene Expression Omnibus (GSE185381). Each matrix was loaded in Seurat with the read10X command, and only genes expressed in more than three cells and cells expressing >500 genes with <15% mitochondrial genes were retained were retained using the CreateSeuratObject command. We extracted erythroid precursor cells from all mononuclear cells in the bone marrow of AML patients and healthy donors, defined as clusters enriched with $CD34^{-}CD36^{+}IL3RA^{-}SLC4A1^{+}GYPA^{+}$ cells.
