# Supplementary material for: Disruption of methionine metabolism drives erythroid cell fate reprogramming by remodeling the H3K4me3 landscape
Source: J Clin Invest. 2026 Jul 14;136(17):e203565. doi: 10.1172/JCI203565 (PMC13528939; doi:10.1172/JCI203565)

Disruption of Methionine Metabolism Drives Erythroid Cell Fate Reprogramming by Remodeling the H3K4me3 Landscape

JCI unedited blot

Full unedited blot for Supplementary Figure 1C

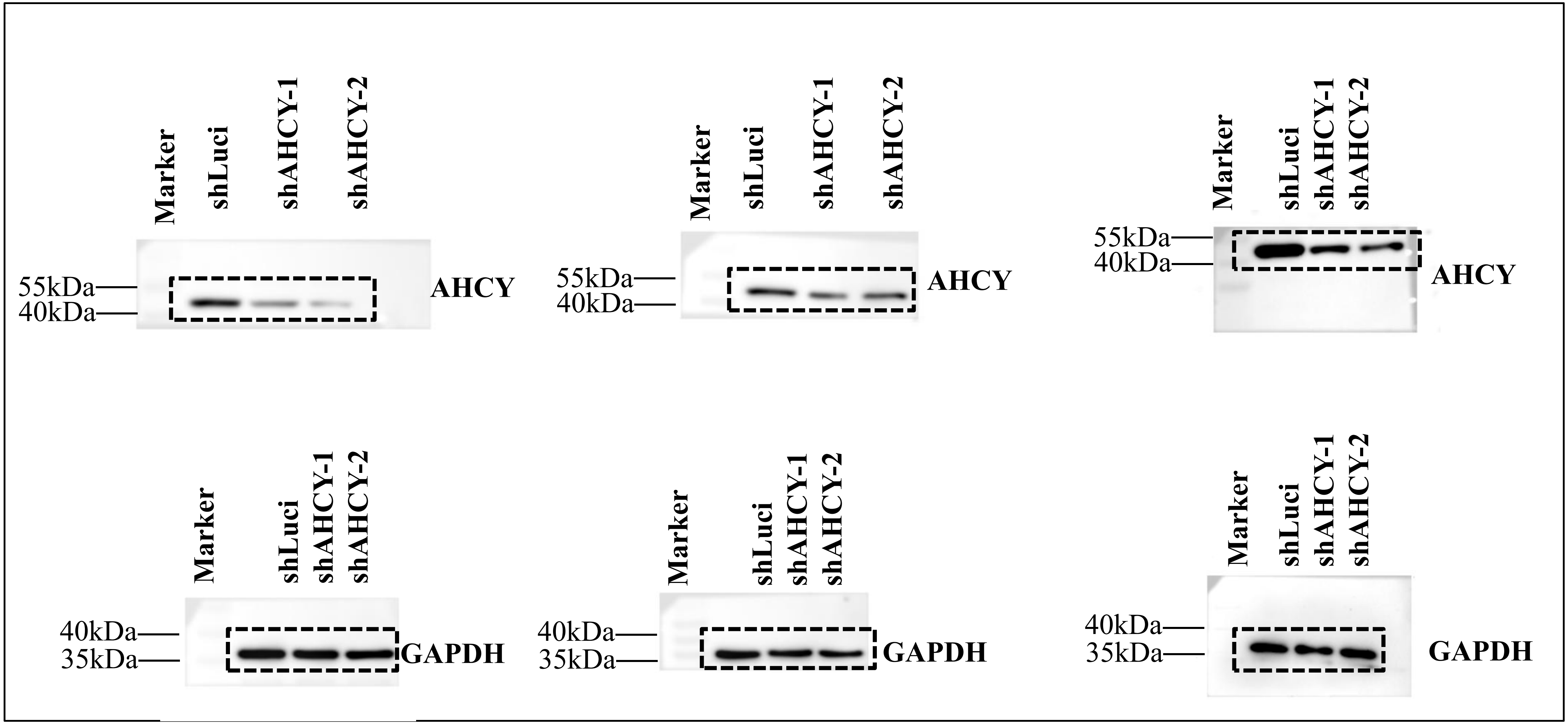

Full unedited blot for Supplementary Figure 1D

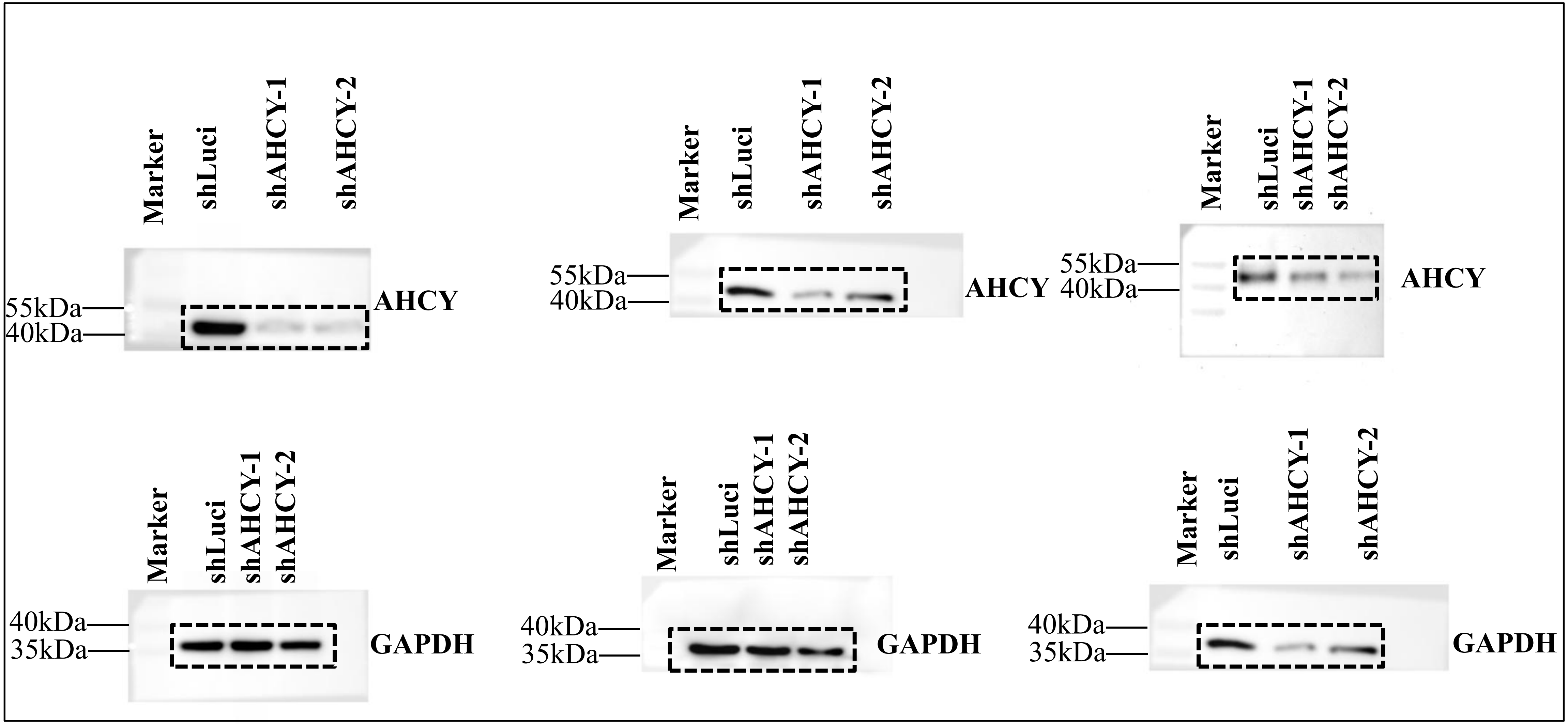

Full unedited blot for Supplementary Figure 3B

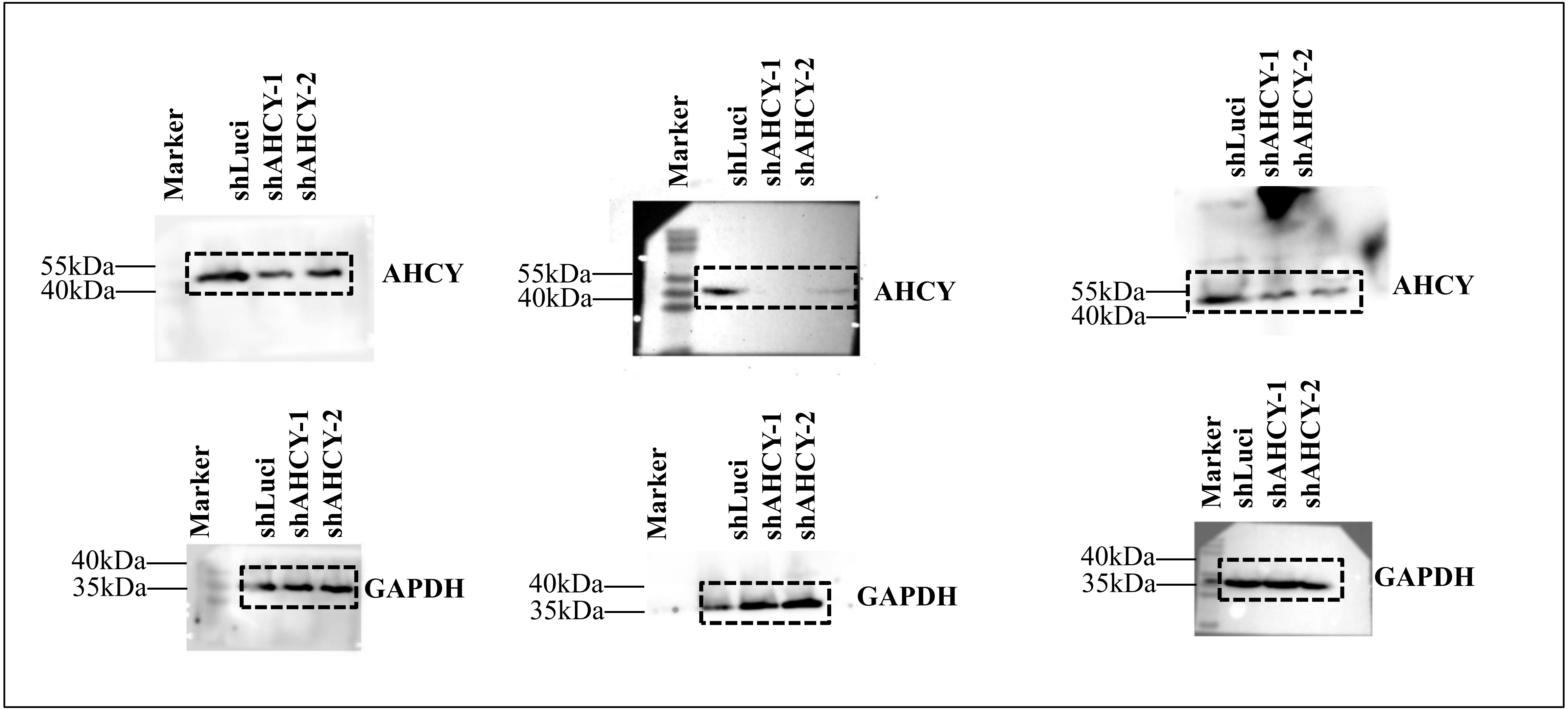

Full unedited blot for Figure 4E

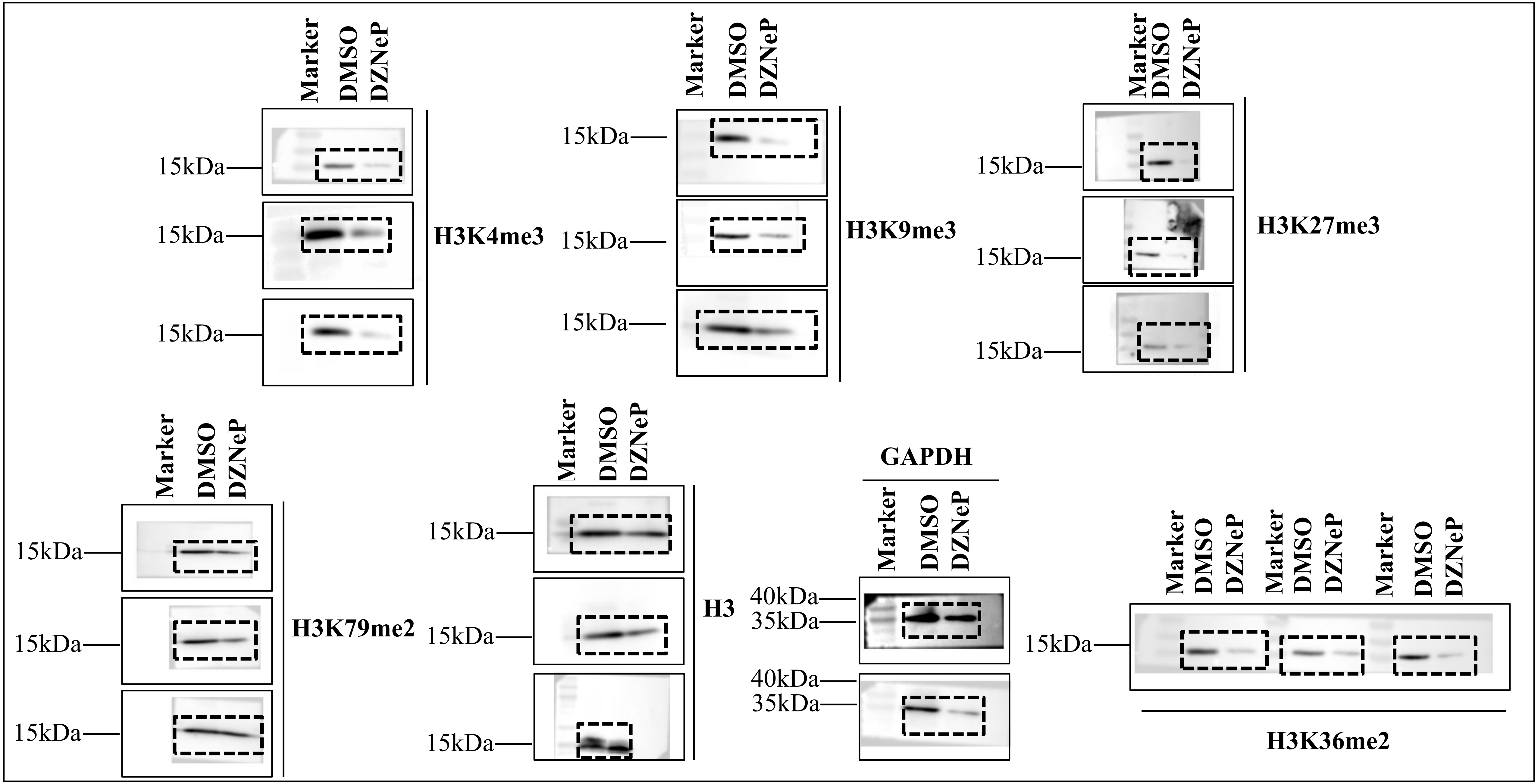

Full unedited blot for Supplementary Figure 8A

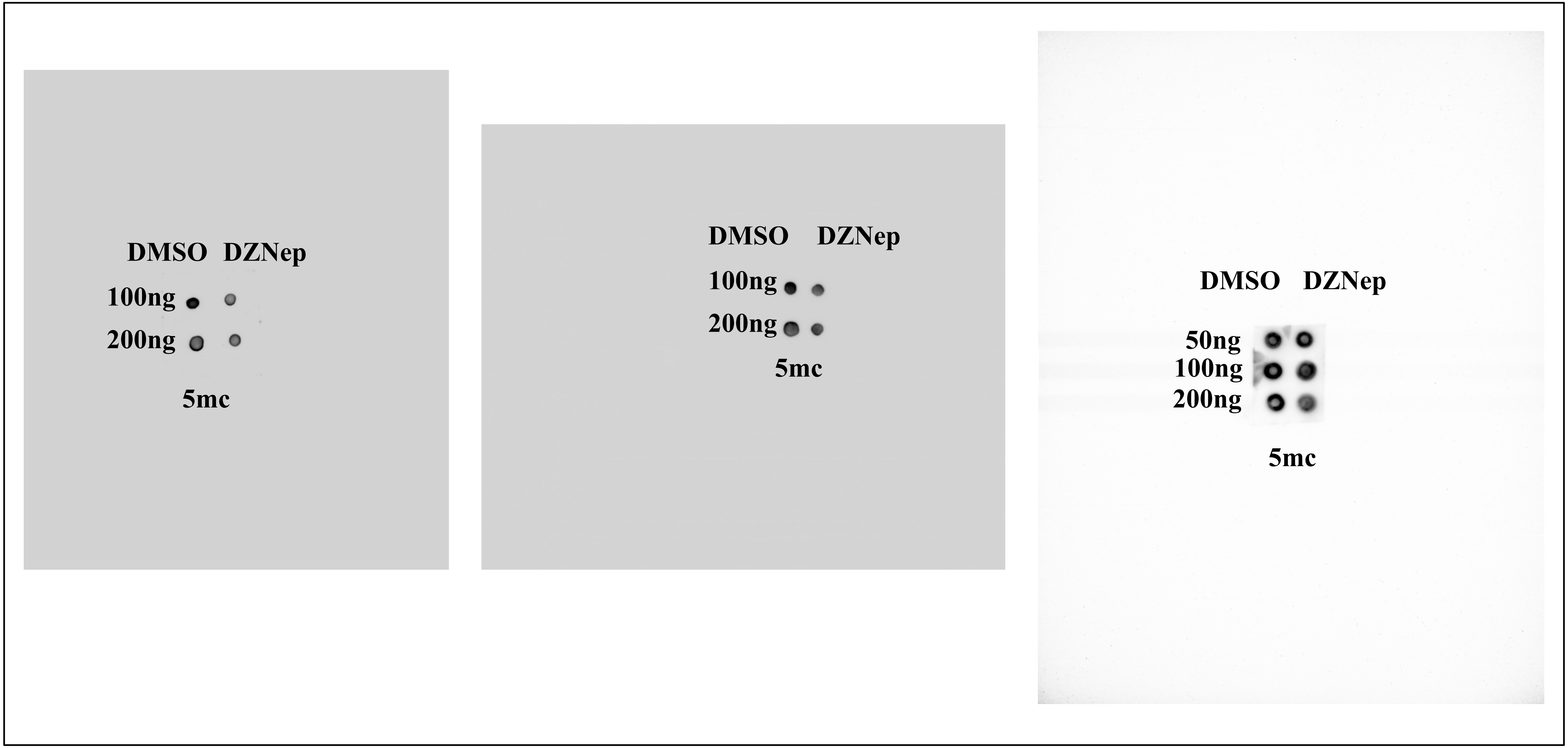

Full unedited blot for Supplementary Figure 8C

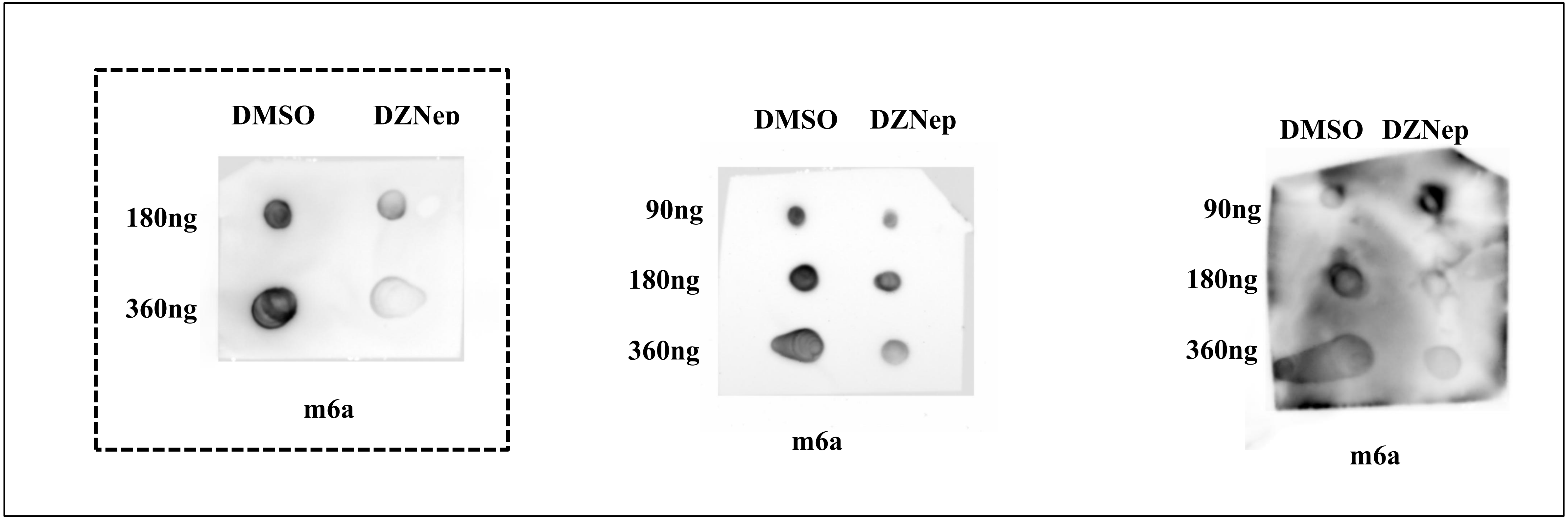

Supplement: Supplementary file 2 [file jci-136-203565-s099.pdf]
